# Broadband Visible Light-Absorbing [70]Fullerene-BODIPY-Triphenylamine Triad: Synthesis and Application as Heavy Atom-Free Organic Triplet Photosensitizer for Photooxidation

**DOI:** 10.3390/molecules26051243

**Published:** 2021-02-25

**Authors:** San-E Zhu, Jian-Hui Zhang, Yu Gong, Li-Feng Dou, Li-Hua Mao, Hong-Dian Lu, Chun-Xiang Wei, Hong Chen, Xue-Fei Wang, Wei Yang

**Affiliations:** 1 School of Energy, Materials and Chemical Engineering, Hefei University, Hefei 230601, China; zhangjianhuiafter@163.com (J.-H.Z.); Huayu202035@163.com (Y.G.); dou_lifeng2010@126.com (L.-F.D.); mlh18970341520@163.com (L.-H.M.); luhdo@hfuu.edu.cn (H.-D.L.); weichunxiang@hfuu.edu.cn (C.-X.W.); chenh113@hfuu.edu.cn (H.C.); 2 School of Chemistry and Chemical Engineering, University of Chinese Academy of Sciences, Beijing 100049, China

**Keywords:** C_70_, BODIPY, triphenylamine, triplet photosensitizer, photooxidation

## Abstract

A broadband visible light-absorbing [70]fullerene-BODIPY-triphenylamine triad (**C_70_-B-T**) has been synthesized and applied as a heavy atom-free organic triplet photosensitizer for photooxidation. By attaching two triphenylmethyl amine units (TPAs) to the π-core of BODIPY via ethynyl linkers, the absorption range of the antenna is extended to 700 nm with a peak at 600 nm. Thus, the absorption spectrum of **C_70_-B-T** almost covers the entire UV–visible region (270–700 nm). The photophysical processes are investigated by means of steady-state and transient spectroscopies. Upon photoexcitation at 339 nm, an efficient energy transfer (ET) from TPA to BODIPY occurs both in **C_70_-B-T** and **B-T**, resulting in the appearance of the BODIPY emission at 664 nm. Direct or indirect (via ET) excitation of the BODIPY-part of **C_70_-B-T** is followed by photoinduced ET from the antenna to C_70_, thus the singlet excited state of C_70_ (^1^C_70_^*^) is populated. Subsequently, the triplet excited state of C_70_ (^3^C_70_^*^) is produced via the intrinsic intersystem crossing of C_70_. The photooxidation ability of **C_70_-B-T** was studied using 1,5-dihydroxy naphthalene (DHN) as a chemical sensor. The photooxidation efficiency of **C_70_-B-T** is higher than that of the individual components of **C_70_-1** and **B-T**, and even higher than that of methylene blue (MB). The photooxidation rate constant of **C_70_-B-T** is 1.47 and 1.51 times as that of **C_70_-1** and MB, respectively. The results indicate that the C_70_-antenna systems can be used as another structure motif for a heavy atom-free organic triplet photosensitizer.

## 1. Introduction

Organic triplet photosensitizers (PSs) have received tremendous attention in recent years due to their wide application in photocatalysis [1,2], photodynamic therapy [3,4,5], and triplet–triplet annihilation upconversion (TTA) [6,7,8]. Broadband visible light-absorbance, hypotoxicity, high efficiency of intersystem crossing (ISC) and long triplet excited state lifetimes are the desired characteristics for triplet PSs [9,10]. However, it is still a challenge to attain the overall properties in one triplet PS.

PSs with heavy atoms can produce the triplet state, whereas the narrow visible light-absorbance, toxicity and high cost limit their applications [11,12,13,14,15]. PSs without heavy atoms usually suffer from the unpredictable ISC ability. In order to address the aforementioned problems, strategies such as using a twisted π-conjugation system [16,17], singlet fission [18,19], spin–orbit charge transfer [20,21], and radicals [22,23] have been developed to enhance the ISC of heavy atom-free PSs. However, the synthetic methods of these compounds are highly demanding. Using fullerene as a spin converter is a useful method to construct PSs with predictable ISC ability, because it possesses an ISC efficiency of unity even after derivatization [24]. Furthermore, fullerene also shows unique properties in biological systems. For example, fullerene exhibits excellent antioxidant [25], antiamyloid [26] and antibacterial [27] properties, has an adhesive and membrane-like potential [28], possesses excellent abilities in penetrating cell membranes and modulating ion transport [29,30], etc. Thus, fullerene-based PSs are promising scaffolds for designing high-technology nanomaterials and drugs in biological field.

C_70_, a higher molecular weight fullerene in the shape of a rugby ball [31], has a much more extended π system and higher absorption in the visible region compared to C_60_ [32]. Like C_60_, C_70_ also possesses high ISC efficiency (near 1.0) and can produce a high quantum yield of ^1^O_2_ (0.81 ± 0.15) [32]. The photodynamic activity and TTA quantum yield of C_70_ are higher than that of the C_60_ counterparts [33,34,35]. In consequence, it is highly promising to synthesize heavy atom-free organic triplet sensitizers with better properties using C_70_ as a spin converter. Similar to C_60_, C_70_ itself is not a good PS due to its low molar extinction coefficient in the visible region. Grafting suitable antennas onto a C_70_ cage seems to be a useful strategy, because significantly greater photosensitization efficiency has been achieved in C_60_-based triplet PSs due to improved visible light absorption [36,37,38,39,40]. Until now, most of the reported studies on the photosensitization of fullerenes have mainly been focused on C_60_. C_70_-antenna systems as triplet photosensitizers have rarely been reported [41].

**Figure 1:**
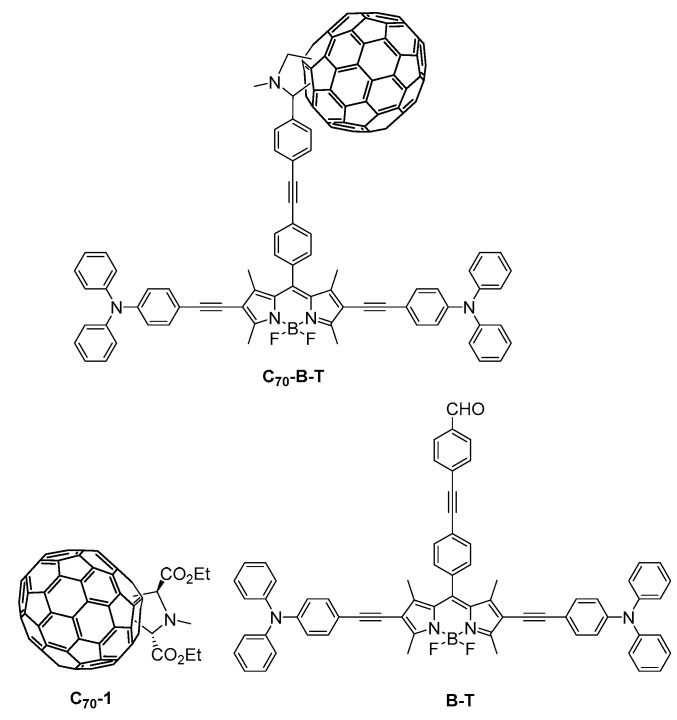
The structures of compounds [70]fullerene-BODIPY-triphenylamine triad (**C_70_-B-T)**, **C_70_-1** and **B-T**.

Herein, we designed and synthesized a broadband visible light-absorbing [70]fullerene-BODIPY-triphenylamine triad (**C_70_-B-T**) as a heavy atom-free organic triplet photosensitizer for photooxidation. BODIPY, owing to its high chemical stability, simple synthetic route and adjustable π-conjugation framework [42,43,44,45,46,47,48,49], was selected as the light-harvesting antenna in this triad. As we know, the absorption wavelength of unsubstituted BODIPY is at ~500 nm and the absorption range is relatively narrow. Therefore, the triphenylmethyl amine (TPA) units were grafted at the “2” and “6” positions of the BODIPY core to improve the absorption range. Direct carbon–carbon coupling at the “2” and “6” positions of the BODIPY will weaken the electronic conjugation and bend the molecule due to steric hindrance [50]. Thus, in this work, alkyne linkers were inserted between the TPA units and BODIPY core to release the steric congestion. In order to place C_70_ and antenna at a fixed distance, TPA-fused BODIPY (**B-T**) and C_70_ were connected by a rigid phenyl acetylene bridge via the Prato reaction. The structures of **C_70_-B-T**, **B-T** and **C_70_-1** are shown in Figure 1. Both **C_70_-B-T** and **B-T** show broadband visible light-absorbance from 270 to 700 nm, covering almost the entire UV–visible region.

## 2. Results and Discussion

### 2.1. Synthesis

**Scheme 1:**
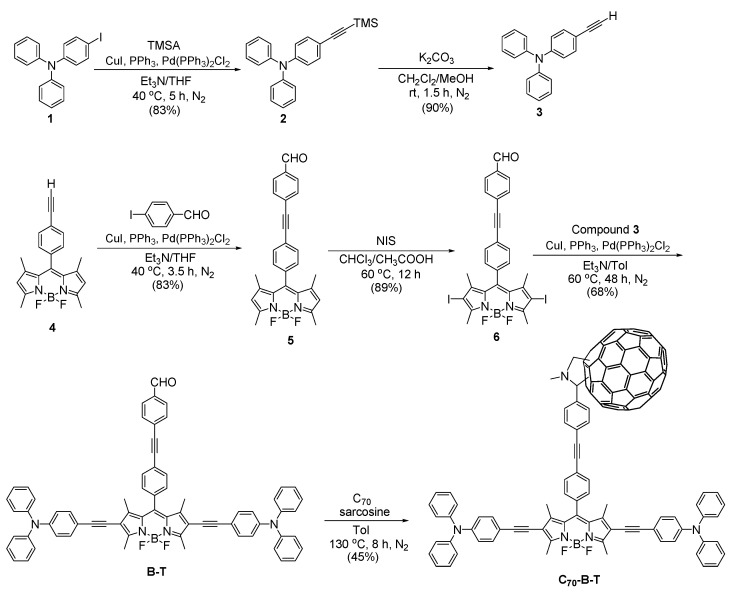
Synthetic procedures for **C_70_-B-T** and **B-T**.

The synthetic procedures of **C_70_-B-T** and **B-T** are outlined in Scheme 1, and the details are given in the Materials and Methods section.

By coupling with aromatic compounds at the “2” and “6” positions, the π-conjugation framework of BODIPY could be extended. In order to construct TPA-fused BODIPY, the preparation of 4-ethynyl-*N*,*N*-diphenylaniline (**3**) and 2,6-diiodo-BODIPY (**6**) was required. Compound **3** was synthesized by a standard Sonogashira reaction between trimethylsilylacetylene (TMSA) and 4-iodo-*N*,*N*-diphenylaniline, followed by deprotection of the trimethylsilyl group.

BODIPY **4** was synthesized according to the reported procedures [36]. The cross-coupling reaction of **4** with 4-iodobenzaldehyde afforded **5** in 83% yield. BODIPY **6** was prepared in 89% yield by treating **5** with *N*-iodosuccinimide (NIS) in the presence of CH_3_CO_2_H. A subsequent double Sonogashira coupling reaction of **6** with **3** afforded **B-T** in 68% yield. Finally, **C_70_-B-T** was obtained in 45% yield by treating **B-T** with C_70_ and sarcosine under nitrogen atmosphere in toluene. The reference compound **C_70_-1** was prepared according to the Prato procedure [51] as described in the Materials and Methods section.

**Figure 2:**
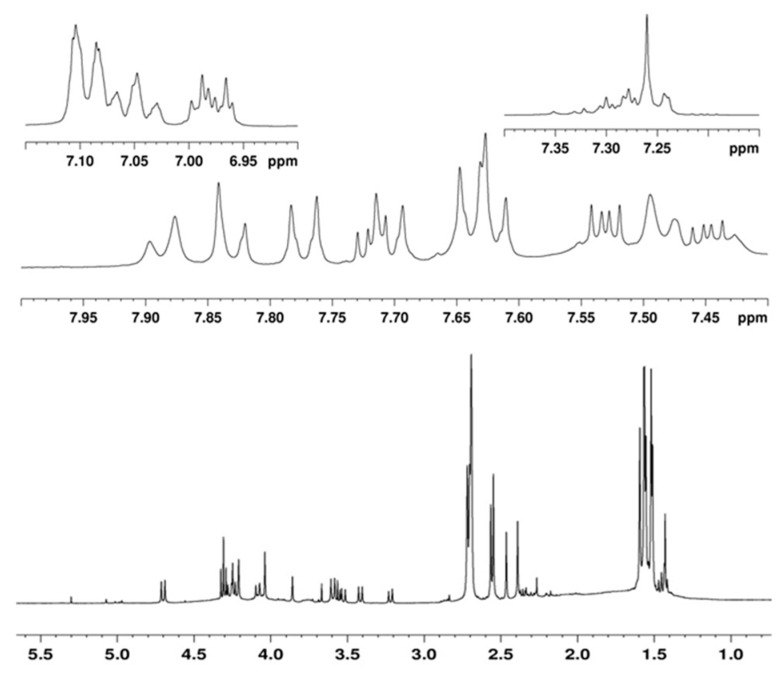
Expansions of the ^1^H-NMR spectrum of **C_70_-B-T** in CDCl_3_.

The structures of all the intermediates and final compounds were fully confirmed by NMR, mass and IR techniques. Due to the lower structural symmetry of C_70_, **C_70_-B-T** and **C_70_-1** are mixture of isomers. The NMR spectra of all the compounds are given in the Supplementary Materials. For the sake of clarity, the expansion of the ^1^H-NMR spectrum of **C_70_-B-T** in CDCl_3_ is shown in Figure 2.

The ^1^H-NMR spectrum shows that there are more than three isomers in **C_70_-B-T** and gives all the expected proton signals. For instance, the peaks at ~7.90–6.96 ppm assign to the phenyl ring protons; peaks at ~5.30–2.39 ppm assign to the pyrrolidine protons, protons of N-C*H*_3_ and pyrrole ring C*H*_3_ and peaks at ~1.59–1.43 ppm assign to the protons of pyrrole ring C*H*_3_. The ^13^C-NMR spectrum of **C_70_-B-T** also shows the expected signals. For example, the peaks from 122–151 ppm assign to the sp^2^-C of C_70_ and benzene ring, peaks at ~80–117 ppm are carbons of alkyne, peaks from 58–71 ppm assign to the sp^3^-C of C_70_ and the carbons of pyrrolidine. The mass spectrum of **C_70_-B-T** gives a molecular peak at *m*/*z* 1854.4471, which is consistent with the calculated data.

### 2.2. UV–Vis Absorption and Steady-State Fluorescence

**Figure 3:**
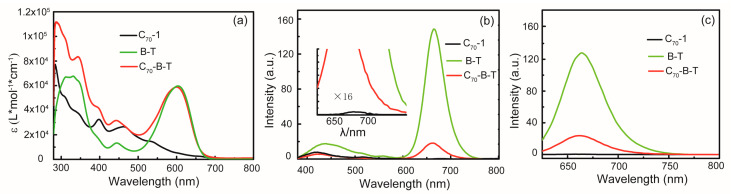
(**a**) UV–vis absorption spectra of **C_70_-1**, **B-T** and **C_70_-B-T** in toluene (c = 1.0 × 10^−5^ mol/L). (**b**) Fluorescence of **C_70_-1**, **B-T** and **C_70_-B-T** in toluene (excited at 339 nm, c = 1.0 × 10^−5^ mol/L). (**c**) Fluorescence of **C_70_-1**, **B-T** and **C_70_-B-T** in toluene (excited at 605 nm, c = 1.0 × 10^−5^ mol/L).

The UV–vis absorption and steady-state fluorescence of **C_70_-B-T**, and the reference compounds **C_70_-1** and **B-T** were recorded in toluene in 1.0 × 10^−5^ M and are shown in Figure 3. Triad **C_70_-B-T** and dyad **B-T** show broadband absorption in the entire UV–visible region (270–700 nm) with a strong absorption peak at about 600 nm (ε = 58,804 L mol^−1^ cm^−1^). Compared with traditional BODIPY, a bathochromic shift of about 100 nm of the low-energy absorption peak is found in **B-T**, indicating that the TPA units extend the conjugation length effectively. The UV–vis spectrum of **C_70_-B-T** is the sum of **C_70_-1** and **B-T** (the data are given in Table 1), suggesting no significant electronic communication between C_70_ and the antenna at ground state.

The steady-state fluorescence spectra of **C_70_-B-T**, **C_70_-1** and **B-T** in toluene upon excitation at 339 and 605 nm are presented in Figure 3b and 3c, respectively. The emission maxima and fluorescence quantum yields of **C_70_-B-T** and **B-T** are listed in Table 1. When the TPA part was excited at 339 nm, only emissions of BODIPY moiety are observed both in **B-T** and **C_70_-B-T**. The fluorescence of TPA (447 nm) part is largely quenched, showing that efficient excitation energy transfer from TPA to BODIPY occurs [52]. The emission peaks of both the triad and dyad are located at 664 nm, but the emission intensity of **C_70_-B-T** is relatively weak compared with that of **B-T** (the quenching efficiency is 88%). Direct excitation of the BODIPY moiety of **C_70_-B-T** and **B-T** at 605 nm results in fluorescence spectra resemble those ones obtained upon TPA-part excitation. **B-T** still gives intense fluorescence with an emission peak at 664 nm (Φ_F_ = 0.22), whereas the emission of **C_70_-B-T** is largely quenched (82%, Φ_F_ = 0.04) due to intramolecular energy or electron transfer.

**Figure 4:**
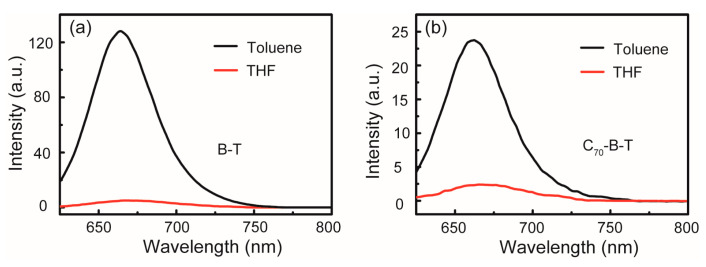
Emission of (**a**) **B-T** and (**b**) **C_70_-B-T** in THF (redline) and toluene (black curve) (1 × 10^−5^ mol/L, exited at 605 nm).

The emissions of **C_70_-B-T** and **B-T** in THF were also measured to investigate the effect of solvent polarity on the emission behavior. The results are shown in Figure 4. The emission intensities of **C_70_-B-T** and **B-T** are quite sensitive to solvent polarity due to the presence of dipole moments inside the molecules. For instance, the emission intensities drop largely in THF in comparison to that in toluene. Whereas the emission peak positions of both **C_70_-B-T** and **B-T** do not show an obvious shift. The low solvent polarity effect suggests the formation of a neutral excited state in **C_70_-B-T** [37,54]. Thus, the emission quenching of the BODIPY part observed in **C_70_-B-T** should mainly be ascribed to the intramolecular energy transfer from **B-T** to C_70_, and the electron transfer from the antenna to C_70_ is not significant [41,55]. The intramolecular energy transfer from **B-T** to C_70_ is possible, because the energy of the S_1_ state of **B-T** is higher than that of C_70_ [32,41]. In the magnified spectrum of Figure 3b, weak fluorescence emissions of C_70_ at 687 nm, 702 nm and 710 nm are observed. However, these emission bands are not observed in **C_70_-B-T** because of the fluorescence overlap.

### 2.3. Time-Resolved Fluorescence Spectroscopy

**Figure 5:**
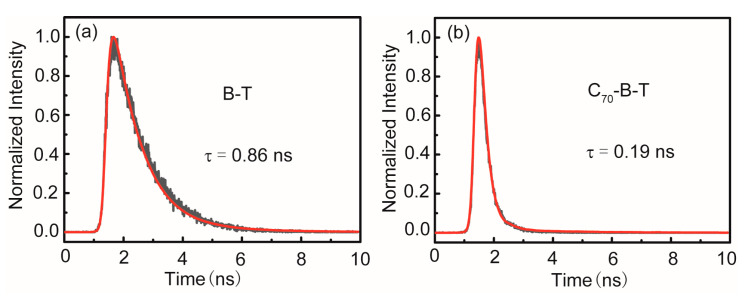
Fluorescence decay traces obtained with a time-correlated single photon counting (TCSPC) of (**a**) **B-T** and (**b**) **C_70_-B-T** in toluene. Excited at 510 nm and recorded at 664 nm.

For deeper insight into the photoinduced intramolecular transfer processes, the fluorescence decays of **C_70_-B-T** and **B-T** were investigated using time-resolved fluorescence spectroscopy techniques. The results are shown in Figure 5.

When excited at 510 nm and recorded the fluorescence signal at 664 nm, **B-T** gives monoexponential decay with fluorescence lifetime of 0.86 ns. Similar results are obtained when **C_70_-B-T** is excited, but the fluorescence lifetime of **C_70_-B-T** is reduced to 0.19 ns, shorter than that of **B-T**. The much shorter fluorescence lifetime of BODIPY part detected in **C_70_-B-T** indicates an effective excitation energy quenching of the BODIPY part by C_70_, which is consistent with the result obtained in the steady-state fluorescence spectra.

The efficiency of energy transfer from BODIPY to C_70_ in **C_70_-B-T** can be calculated by using Equation (1).

(1) $$\Phi_{1}=\frac{k_{1}}{\left(k_{0\;}+{\;k}_{1}\right)}=\;\frac{1/\tau_{1}-1/\tau_{0}}{1/\tau_{1}}$$

where τ_0_ and τ_1_ are the fluorescence lifetimes of **B-T** and **C_70_-B-T**, respectively. Thus, the energy transfer efficiency Φ_1_ is determined as 0.78 for **C_70_-B-T**, which is in consistence with the result of the steady-state fluorescence spectra

### 2.4. Nanosecond Time-Resolved Transient Absorption Spectroscopy

**Figure 6:**
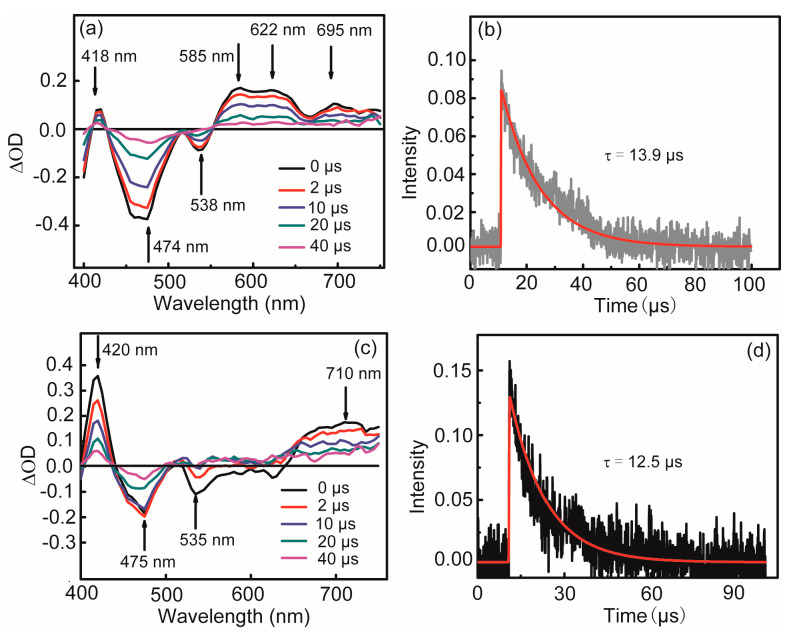
(**a**) Nanosecond time-resolved transient absorption spectrum of **C_70_-B-T** upon excitation at 532 nm with a pulse laser (7 ns, 1 Hz) in deaerated toluene at room temperature. (**b**) Decay of **C_70_-B-T** at 700 nm. (**c**) Nanosecond time-resolved transient absorption spectrum of **C_70_-1** upon excitation at 532 nm with a pulse laser (7 ns, 1 Hz) in deaerated toluene at room temperature. (**d**) Decay of **C_70_-1** at 700 nm. Arrows indicate the spectral trend with time increasing.

Nanosecond time-resolved transient absorption spectroscopy was used to investigate the triplet excited states of the triad and **C_70_-1**. The nanosecond transient absorption spectra were recorded by a nanosecond flash photolysis system (LP980 ENDINBURGH) with a pulse laser (7 ns, 1 Hz) from a Neodymium-doped Yttrium Aluminium Garnet (Nd:YAG) laser at a wavelength of 532 nm. The results are shown in Figure 6.

Upon excitation at 532 nm, bleaching at about 474 and 538 nm was observed for both **C_70_-B-T** and **C_70_-1** due to ground state absorption of C_70_. **C_70_-1** shows a sharp transient absorption band at 420 nm and a broad transient absorption band from 644 to 735 nm (Figure 6c). In the time-resolved transient absorption spectrum of **C_70_-B-T**, no bleaching of the steady-state absorption of **B-T** part is observed. **C_70_-B-T** also shows a characteristic absorption band of ^3^C_70_* at 418 nm. However, the broad transient absorption band of **C_70_-B-T** is blue-shifted and splits into three bands with peaks at ~585, 622 and 695 nm due to the derivatization of C_70_ by the antenna [41]. The dynamic decay behaviors of the three bands are similar to that of the peak at 418 nm, suggesting that the three bands also belong to the absorptions of ^3^C_70_*, agreed well with the reported studies [41]. Therefore, the peaks observed in the transient absorption spectrum of **C_70_-B-T** should be ascribed to the absorptions of the ^3^C_70_*. When excited, the triplet state of **C_70_-B-T** is exclusively localized on the C_70_ unit. The lifetime of the triplet state of **C_70_-B-T** is 13.9 µs, slightly longer than that of **C_70_-1** (12.5 µs).

### 2.5. Photooxidation of 1,5-Dihydroxy Naphthalene Mediated by ^1^O_2_

**Figure 7:**
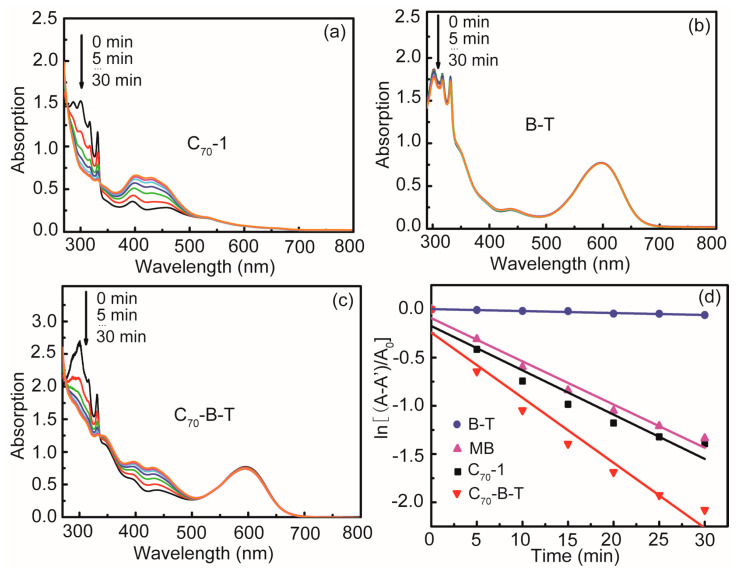
Absorption spectral evolution for the photooxidation of DHN using (**a**) **C_70_-1**, (**b**) **B-T** and (**c**) **C_70_-B-T** as sensitizers. (**d**) Plots of ln[(A − A′)/A_0_] vs. irradiation time (t) for the photooxidation of DHN using different sensitizers (collected at 301 nm). c[sensitizers] = 1.0 × 10^−5^ mol L^−1^, c[DHN] = 1.0 × 10^−4^ mol L^−1^. In CH_2_Cl_2_/MeOH (9:1, *v*/*v*). A, A′, and A_0_ were the absorbances at 301 nm, where A was the absorption of DHN and sensitizer, A′ was the absorption of sensitizer, A_0_ was the initial absorption of DHN.

The photooxidation ability of **C_70_-B-T** was studied by using 1,5-dihydroxy naphthalene (DHN) as a ^1^O_2_ scavenger. In the presence of ^1^O_2_, DHN can be easily oxidized to juglone [56]. The kinetics of the photooxidation could be measured by following the decrease in the absorption of DHN at 301 nm or the increase in the absorption of juglone at 427 nm with time. The spectral responses of DHN using **C_70_-B-T**, **B-T**, **C_70_-1**, and methylene blue (MB) as the sensitizers upon broadband excitation with a xenon lamp are presented in Figure 7 and Figure S1, respectively. For **C_70_-B-T**, **C_70_-1**, and MB, the change of the absorption at 301 nm is obvious, indicating the significant consumption of DHN and the efficient photosensitization ability of the triplet PSs, whereas, nearly no UV–vis absorption change is observed in the spectral responses of DHN with **B-T** as the photosensitizer. The photostability of **C_70_-B-T** was also investigated by exposing to light for 1 h and no decrease is observed in the absorption (Figure S2). This further proves that the decrease in the absorption at 301 nm is caused by photooxidation instead of the decomposition of the photosensitizers.

The photooxidation ability of the triplet photosensitizers was quantitatively compared by plotting the ln[(A − A′)/A_0_] against the irradiation time. The photooxidation rate constant and the yield of singlet oxygen (Φ_Δ_) of the photosensitizers were calculated [37,57], and the data are listed in Table 2.

Among all the investigated photosensitizers, **C_70_-B-T** gives the highest photooxidation efficiency. The photooxidation rate constant (k_obs_) of **C_70_-B-T** is 67.5 × 10^−3^ min^−1^, which is 1.47 times as that of **C_70_-1** (45.9 × 10^−3^ min^−1^) and 1.51 times as that of MB (44.8 × 10^−3^ min^−1^). **B-T** does not show obvious photooxidation ability. The more efficient photooxidation of **C_70_-B-T** compared with that of **C_70_-1** demonstrates that the broadband visible light absorption antenna improves the photosensitizing ability. Here, the enhanced photosensitizing ability of **C_70_-B-T** compared with that of **C_70_-1** and **B-T** should be attributed to the synergetic effect of C_70_ and **B-T**. First, **B-T** harvested a broadband visible light and be excited to its excited singlet easily, then efficient intramolecular energy transfer from **B-T** to C_70_ occurred and formed the ^1^C_70_*, finally the highly efficient ISC of C_70_ would eventually lead to the population of ^3^C_70_*. These photophysical processes can be supported by the steady-state and transient data mentioned above.

## 3. Materials and Methods

### 3.1. Materials

All reagents were obtained from commercial sources. C_70_, ethyl 2-(methylamino)acetate hydrochloride, ethyl glyoxylate, 4-iodo-*N*,*N*-diphenylaniline, cuprous iodide (CuI), triphenylphosphine (PPh_3_), trimethylsilylacetylene (TMSA), potassium carbonate (K_2_CO_3_), anhydrous sodium sulphate (Na_2_SO_4_), *N*-iodosuccinimide (NIS), sodium thiosulfate (Na_2_S_2_O_3_) and DHN were purchased from Alfa Aesar. Trans-dichlorobis(triphenyl-phosphine)Palladium(II) (Pd(PPh_3_)_2_Cl_2_) was purchased from Shanxi Kaida Chemical engineering Co., Ltd. Sarcosine, 4-iodobenzaldehyde and 1,2-dichlorobenzene (ODCB) were purchased from J&K Scientific LTD (Beijing, China).

Silica gel, carbon disulfide (CS_2_), dichloromethane (DCM), petroleum ether (PE), methanol (MeOH), tetrahydrofuran (THF), triethylamine (Et_3_N), acetic acid (CH_3_COOH), trichloromethane (CHCl_3_), and toluene were purchased from Sinopharm Chemical Reagent Co., Ltd (Shanghai, China). THF was distilled over sodium and benzophenone, other reagents used for the synthesis were used directly.

All synthesis compounds were characterized by ^1^H and ^13^C-NMR spectroscopy on a BRUKER 400 MHz spectrometer. The mess analyses were performed using a Bruker ultrafleXtreme MALDI TOF/TOF (Bremen, Germany).

### 3.2. Synthesis

#### 3.2.1. Synthesis of **C_70_-1**

The solution of C_70_ (140 mg, 0.16 mmol), ethyl 2-(methylamino)acetate hydrochloride (49.1 mg, 0.32 mmol) in ODCB (15 mL) was bubbled with N_2_ for 30 min at room temperature. Ethyl glyoxylate (158 µL, 0.8 mmol, in a 50% toluene solution) was added and the reaction mixture was stirred at 130 °C for 90 min monitored by thin layer chromatography (TLC). After the solvent was removed by reduced pressure, the mixture was purified by silica gel column chromatography using CS_2_/DCM (2/1, *v*/*v*) as the eluent to give **C_70_-1** as a brown black powder (70.0 mg, 42%). UV-vis (toluence) λ_max_/nm 284 (77230 L mol^−1^ cm^−1^), 307 (51491 L mol^−1^cm^−1^), 398 (32516 L mol^−1^ cm^−1^), 462 (26537 L mol^−1^ cm^−1^), 537 (14119 L mol^−1^cm^−1^), 666 (2426 L mol^−1^cm^−1^). ^1^H-NMR (400 MHz, CDCl_3_) *δ* 5.83 (s, C*H*CO_2_), 5.35 (s, C*H*CO_2_), 5.30 (s, C*H*CO_2_), 4.81(s, C*H*CO_2_), 4.63–4.37(m, OC*H*_2_CH_3_), 4.32–4.17(m, OC*H*_2_CH_3_), 4.15-3.99 (m, OC*H*_2_CH_3_), 3.10 (s, NC*H*_3_), 2.83 (s, NC*H*_3_), 2.68 (s, NC*H*_3_), 2.62 (s, NC*H*_3_), 1.54 (t, *J* = 7.1 Hz, OCH_2_C*H*_3_), 1.42 (t, *J* = 7.2 Hz, OCH_2_C*H*_3_), 1.17 (t, *J* = 7.1 Hz, OCH_2_C*H*_3_), 1.04 (t, *J* = 7.2 Hz, OCH_2_C*H*_3_). ^13^C-NMR (100 MHz, CDCl_3_) *δ* 170.18, 170.06, 157.73, 155.60, 155.32, 154.87, 151.56, 151.20, 151.14, 150.89, 150.83, 150.50, 150.46, 150.01, 149.78, 149.46, 149.37, 149.23, 149.20, 149.06, 148.87, 148.83, 147.60, 147.54, 147.29, 147.25, 147.18, 147.14, 147.12, 147.06, 146.98, 146.32, 146.10, 145.99, 145.91, 143.52, 143.50, 143.48, 143.40, 143.21, 141.42, 140.74, 140.46, 140.23, 138.52, 137.64, 133.90, 133.70, 133.27, 131.86, 131.73, 131.53, 131.49, 131.36, 75.93, 72.28, 65.54, 64.58, 62.07, 61.65, 35.18, 29.86, 14.84, 14.50, 14.70, 14.35, 14.28. FT-IR υ/cm^−1^ (KBr) 2922, 2851, 1746, 1733, 1632, 1427, 1369, 1340, 1190, 1131, 1052, 795, 671, 637, 580, 534. HRMS (MALDI-TOF) *m/z* calcd for C_79_H_15_NO_4_[M^−•^]1041.1007, found 1041.1001.

#### 3.2.2. Synthesis of Compound **2**

4-Iodo-*N*,*N*-diphenylaniline **1** (500.0 mg, 1.35 mmol), CuI (128.3 mg, 0.67 mmol) and PPh_3_ (176.6 mg, 0.67 mmol) were dissolved in 20 mL of THF/Et_3_N (1/3, *v*/*v*) in an oven-dried, 50 mL one neck flask equipped with a gas inlet adaptor. The mixture was stirred at room temperature under N_2_ atmosphere for 30 min. Then Pd(PPh_3_)_2_Cl_2_ (189.0 mg, 0.27 mmol) and TMSA (945 µL, 6.74 mmol) were added. The mixture was stirred at 40 °C under N_2_ atmosphere for 5 h until TLC monitoring indicated the disappearance of compound **1**. The solvent was removed in vacuo, and the crude reaction mixture was purified by silica gel column chromatography using PE/CH_2_Cl_2_ (2/1, *v*/*v*) as eluent to give **2** [58] in 83% yield (391.0 mg). ^1^H-NMR (400 MHz, CDCl_3_) *δ* 7.34 (d, *J* = 8.7 Hz, 2H), 7.31-7.27 (m, 4H), 7.12–7.06 (m, 6H), 6.98 (d, *J* = 8.7 Hz, 2H), 0.27 (s, 9H).

#### 3.2.3. Synthesis of Compound **3**

Compound **2** (120.0 mg, 0.35 mmol) was dissolved in 7 mL of CH_2_Cl_2_/MeOH (1/1, *v*/*v*) in an oven-dried, 25 mL one neck flask equipped with a gas inlet adaptor. The mixture was stirred at room temperature under N_2_ atmosphere for 30 min. Then, K_2_CO_3_ (145.5 mg, 1.05 mmol) was added. The mixture was stirred at room temperature under N_2_ atmosphere for 1.5 h until TLC monitoring indicated the disappearance of compound **2**. The crude mixture was extracted with CH_2_Cl_2_ and H_2_O three times. The organic layer was dried by Na_2_SO_4_ and the solvent was then removed. The crude product was purified by silica gel column chromatography using PE/CH_2_Cl_2_ (2/1, *v*/*v*) as eluent to give **3** [58] in 90% yield (85.8 mg). ^1^H-NMR (400 MHz, CDCl_3_) *δ* 7.33 (d, *J* = 8.7 Hz, 2H), 7.29–7.25 (m, 4H), 7.11–7.04 (m, 6H), 6.97 (d, *J* = 8.6 Hz, 2H), 3.02 (s, 1H).

#### 3.2.4. Synthesis of Compound **5**

Compound **4** was synthesized by adapting the literature procedure and used directly [36]. **4** (394.4 mg, 1.13 mmol), 4-iodobenzaldehyde (313.2 mg, 1.35 mmol), CuI (107.6 mg, 0.57 mmol) and PPh_3_ (148.2 mg, 0.57 mmol) were dissolved in 24 mL of THF/Et_3_N (1/2, *v*/*v*) in an oven-dried, 50 mL one-neck flask equipped with a gas inlet adaptor. The mixture was stirred at room temperature under N_2_ atmosphere for 30 min. Then, Pd(PPh_3_)_2_Cl_2_ (158.6 mg, 0.23 mmol) was added. The mixture was stirred at 40 °C under N_2_ atmosphere for 3.5 h until TLC monitoring indicated the disappearance of **4**. The solvent was removed in vacuo, and the crude reaction mixture was purified by silica gel column chromatography using PE/CH_2_Cl_2_ (4/1, *v*/*v*) as eluent to give **5** in 83% yield (425.4 mg). ^1^H-NMR (400 MHz, CDCl_3_) *δ* 10.04 (s, 1H), 7.90 (d, *J* = 8.2 Hz, 2H), 7.72–7.69 (m, 4H), 7.33 (d, *J* = 8.2 Hz, 2H), 6.00 (s, 2H), 2.56 (s, 6H), 1.43 (s, 6H). ^13^C-NMR (100 MHz, CDCl_3_) *δ* 191.52, 156.02, 143.05, 140.63, 135.85, 135.80, 132.64, 132.31, 131.26, 129.80, 129.19, 128.53, 123.47, 121.57, 92.61, 89.91, 14.75. FT-IR υ/cm^−1^ (KBr) 2923, 2852, 1698, 1600, 1542, 1510, 1468, 1436, 1402, 1369, 1304, 1260, 1193, 1156, 1120, 1082, 1050, 1019, 979, 830, 766, 707, 477. HRMS (MALDI-TOF) *m*/*z* calcd for C_28_H_23_BF_2_N_2_O[M^+•^]452.1871, found 452.1876.

#### 3.2.5. Synthesis of Compound **6**

Compound **5** (200.1 mg, 0.44 mmol) and NIS (217.7 mg, 0.97 mmol) were dissolved in 12 mL of CHCl_3_/CH_3_COOH (2/1, *v*/*v*) in a 25 mL one-neck flask and the mixture was stirred at 60 °C for 12 h, monitored by TLC. Then the mixture was extracted with CH_2_Cl_2_ and a saturated sodium thiosulfate aqueous solution three times. The combined organic layer was dried with anhydrous Na_2_SO_4_, filtered and concentrated. The crude reaction mixture was subjected to silica gel column chromatography using PE/CH_2_Cl_2_ (2/1, *v*/*v*) as eluent to give **6** in 89% yield (280.0 mg). ^1^H-NMR (400 MHz, CDCl_3_) *δ* 10.05 (s, 1H), 7.91 (d, *J* = 8.2 Hz, 2H), 7.73 (d, *J* = 8.2 Hz, 2H), 7.72 (d, *J* = 8.1 Hz, 2H), 7.30 (d, *J* = 8.2 Hz, 2H), 2.66 (s, 6H), 1.45 (s, 6H). ^13^C-NMR (100 MHz, CDCl_3_) *δ* 191.50, 157.37, 145.26, 140.27, 135.90, 135.45, 132.91, 132.36, 131.15, 129.82, 129.03, 128.37, 124.15, 92.28, 90.37, 86.07, 17.36. FT-IR υ/cm^−1^ (KBr) 2923, 1690, 1599, 1544, 1530, 1489, 1439, 1399, 1345, 1307, 1204, 1181, 1113, 1089, 995,918, 823, 765, 706, 588, 525. HRMS (MALDI-TOF) *m*/*z* calcd for C_28_H_21_BF_2_I_2_N_2_O[M^+•^]703.9804, found 703.9809.

#### 3.2.6. Synthesis of Compound **B-T**

Compound **6** (100.0 mg, 0.14 mmol), CuI (13.5 mg, 0.07 mmol) and PPh_3_ (18.6 mg, 0.07 mmol) were dissolved in 12 mL of toluene/Et_3_N (1/3, *v*/*v*) in an oven-dried, 25 mL one neck flask equipped with a gas inlet adaptor. The mixture was stirred at room temperature under N_2_ atmosphere for 30 min. Then Pd(PPh_3_)_2_Cl_2_ (19.9 mg, 0.03 mmol) and compound **3** (152.8 mg, 0.57 mmol) were added. The mixture was stirred at 60 °C under N_2_ atmosphere for 48 h until TLC monitoring indicated the disappearance of compound **6**. The solvent was removed in vacuo, and the crude reaction mixture was purified by silica gel column chromatography using PE/CH_2_Cl_2_ (4/1, *v*/*v*) as eluent to give **B-T** in 68% yield (95.4 mg). UV–vis (toluence) λ_max_/nm 330 (67,683 L mol^−1^ cm^−1^), 443 (13,215 L mol^−1^ cm^−1^), 603 (59,703 L mol^−1^ cm^−1^). ^1^H-NMR (400 MHz, CDCl_3_) *δ* 10.04 (s, 1H), 7.90 (d, *J* = 8.4 Hz, 2H), 7.74–7.71 (m, 4H), 7.34 (d, *J* = 8.3 Hz, 2H),7.31–7.28 (m, 6H), 7.27–7.24 (m,6H), 7.11–7.08 (m, 8H), 7.07–7.03 (m, 4H), 6.99–6.97 (m, 4H), 2.71 (s, 6H), 1.56 (s, 6H). ^13^C-NMR (100 MHz, CDCl_3_) *δ* 191.53, 158.87, 148.03, 147.28, 143.25, 141.02, 135.91, 135.38, 132.79, 132.43, 132.35, 131.10, 129.82, 129.53, 129.14, 128.49, 125.06, 123.89, 123.69, 122.51, 116.89, 116.22, 97.05, 92.51, 90.19, 80.69, 13.90, 13.77. FT-IR υ/cm^−1^ (KBr) 2923, 2853, 1635, 1590, 1525, 1492, 1400, 1318, 1264, 1186, 1173, 1078, 1011, 795, 753, 710, 695, 586, 533, 506. HRMS (MALDI-TOF) *m/*z calcd for C_68_H_49_BF_2_N_4_O[M^+•^] 986.3973, found 986.3976.

#### 3.2.7. Synthesis of Compound **C_70_-B-T**

The mixture of C_70_ (35.4 mg, 0.04 mmol), compound **B-T** (25.0 mg, 0.025 mmol) and sarcosine (18.8 mg, 0.21 mmol) was dissolved in 5 mL of toluene and stirred at 130 °C under N_2_ atmosphere for 8 h, monitored by TLC. Then the solvent was removed under reduced pressure. The mixture was subjected to silica gel column chromatography using CS_2_ as the eluent to afford unreacted C_70_, then using CS_2_/CH_2_Cl_2_ (2/1, *v*/*v*) as the eluent to give **C_70_-B-T** as a mixture of isomers (21.3 mg, 45%). UV–vis (toluene) λ_max_/nm 284 (111272 mol^−1^ cm^−1^), 310 (98534 mol^−1^ cm^−1^), 344 (83490 L mol^−1^ cm^−1^), 443 (31724 L mol^−1^ cm^−1^), 600 (58804 L mol^−1^ cm^−1^). ^1^H-NMR (400 MHz, CDCl_3_) *δ* 7.90–7.61 (m, phenyl ring H), 7.54–7.43 (m, phenyl ring H), 7.35–7.19 (m, phenyl ring H), 7.11–7.03 (m, phenyl ring H), 7.00–6.96 (m, phenyl ring H), 5.30 (s, NC*H*Ph), 5.07 (s, NC*H*Ph), 5.01 (s, NC*H*Ph), 4.97 (s, NC*H*Ph), 4.70 (d, *J* = 9.4 Hz, NC*H*_2_), 4.32–4.21 (NC*H*Ph, NC*H*_2_), 4.08 (d, *J* = 9.7 Hz, NC*H*_2_), 4.04 (s, NC*H*Ph), 3.86 (s, NC*H*Ph), 4.67–4.51 (NC*H*Ph, NC*H*_2_), 3.41 (d, *J* = 9.4 Hz, NC*H*_2_), 3.22 (d, *J* = 9.5 Hz, NC*H*_2_), 2.72 (s, NC*H*_3_, pyrrole ring C*H*_3_), 2.70 (s, NC*H*_3_, pyrrole ring C*H*_3_), 2.69 (s, NC*H*_3_, pyrrole ring C*H*_3_), 2.56 (s, NC*H*_3_, pyrrole ring C*H*_3_), 2.55 (s, NC*H*_3_, pyrrole ring C*H*_3_), 2.46 (s, NC*H*_3_, pyrrole ring C*H*_3_), 2.39 (s, NC*H*_3_, pyrrole ring C*H*_3_), 1.59 (s, pyrrole ring C*H*_3_), 1.56 (s, pyrrole ring C*H*_3_), 1.55 (s, pyrrole ring C*H*_3_), 1.52 (s, pyrrole ring C*H*_3_), 1.51 (s, pyrrole ring C*H*_3_), 1.43 (s, pyrrole ring C*H*_3_). ^13^C-NMR (100 MHz, CDCl_3_) *δ* 167.85, 167.53, 158.95, 158.73, 158.50, 158.03, 156.54, 156.21, 155.52, 155.00, 154.96, 154.81, 154.77, 153.49, 152.54, 151.82, 151.66, 151.58, 151.56, 151.52, 151.47, 151.43, 151.21, 151.16, 151.02, 150.95, 150.83, 150.78, 150.76, 150.73, 150.64, 150.85, 150.55, 150.36, 150.32, 150.17, 150.07, 150.01, 149.96, 149.89, 149.83, 149.80, 149.77, 149.49, 149.44, 149.39, 149.34, 149.27, 149.21, 149.18, 149.10, 149.07, 149.05, 148.95, 148.91, 148.89, 148.79, 148.73, 148.43, 148.39, 148.37, 148.33, 148.25, 148.19, 148.15, 147.95, 147.89, 147.83, 147.60, 147.50, 147.24, 147.16, 147.02, 146.97, 146.95, 146.91, 146.75, 146.65, 146.57, 146.39, 146.32, 146.29, 146.23, 145.95, 145.89, 145.83, 145.78, 145.73, 145.65, 145.53, 145.44, 145.25, 145.17, 145.01, 144.87, 144.77, 144.64, 144.52, 144.48, 144.45, 144.39, 143.99, 143.90, 143.81, 143.73, 143.56, 143.51, 143.43, 143.36, 143.32, 143.28, 143.13, 142.99, 142.92, 142.85, 142.81, 142.40, 142.18, 142.08, 142.06, 142.02, 141.75, 141.28, 141.23, 141.15, 141.06, 140.95, 140.88, 140.81, 140.74, 140.67, 140.64, 140.54, 140.44, 140.29, 140.17, 138.75, 138.02, 137.97, 137.75, 137.58, 137.53, 137.42, 134.67, 134.61, 134.58, 133.92, 133.87, 133.75, 133.67, 133.60, 133.07, 132.67, 132.44, 132.11, 131.88, 131.82, 131.71, 131.68, 131.66, 131.60, 131.42, 131.35, 131.30, 131.19, 131.09, 131.05, 129.52, 128.97, 128.38, 128.28, 125.02, 124.57, 124.40, 123.66, 123.13, 122.86, 122.50, 116.87, 116.25, 97.12, 91.25, 91.14, 90.97, 89.61, 89.52, 89.44, 89.35, 88.09, 86.09, 83.15, 82.83, 82.18, 80.84, 79.93, 70.85, 70.27, 69.98, 68.86, 68.27, 66.68, 66.44, 66.28, 65.70, 62.18, 60.46, 58.87, 58.47, 39.69, 39.62, 32.06, 30.69, 30.44, 30.30, 30.18, 29.84, 29.50, 14.28, 13.89, 13.81. FT-IR υ/cm^−1^ (KBr) 2959, 2923, 2852, 1708, 1673, 1592, 1502, 1402, 1339, 1317, 1283, 1174, 1090, 796, 546, 419. HRMS (MALDI-TOF) *m*/*z* calcd for C_21_H_19_BF_2_N_2_ [M^+•^] 1857.4479, found 1854.4471.

### 3.3. Photooxidation Experiment

Compounds **C_70_-B-T**, **C_70_-1**, **B-T** and MB in a concentration of 2.0 × 10^−5^ mol L^−1^ and DHN in a concentration of 2.0 × 10^−4^ mol L^−1^ were dissolved in CH_2_Cl_2_/MeOH (9:1, *v*/*v*), respectively. Then, the above solutions of sensitizers and DHN were mixed in a volume ratio of 1:1, and O_2_ was bubbled through the mixture for 10 min. The mixture was then placed in a quartz cell and irradiated with a broadband light source-xenon lamp using 0.72 M NaNO_2_ aqueous solution as a cutoff filter (0.17 mW/cm^2^). The consumption of DHN was monitored by a decrease in the absorption at 301 nm using a UV–vis spectrophotometer (UV-1800, Mapada, Shanghai, China) at intervals of 5 min.

The singlet oxygen quantum yield (Φ_∆_) was determined by using Equation (2).

(2) $$\Phi_{\Delta }\;=\;\Phi_{\Delta }\;(\mathrm{std})\frac{k_{\mathrm{obs}}\left(x\right)•I(\mathrm{std})}{k_{\mathrm{obs}}\left(\mathrm{std}\right)•I(x)}$$

In the equation, Φ_∆_(std) is the singlet oxygen generation quantum yield of MB (0.57 in CH_2_Cl_2_), k_obs_(x) and k_obs_(std) were the absolute value of the slopes of ln[(A-A′)/A_0_] versus irradiation time for the photooxidation of DHN by sensitizers and MB, respectively. I(x) and I(std) were the total light intensities absorbed by sensitizers and MB, respectively.

### 3.4. Photostability Experiment

**C_70_-B-T** in a concentration of 1.0 × 10^−5^ mol L^−1^ in CH_2_Cl_2_/MeOH (9:1, *v*/*v*) was placed in a quartz cell and irradiated with a xenon lamp (0.17 mW/cm^2^) continuously for 1 h. The spectral response of **C_70_-B-T** was recorded using a UV–vis spectrophotometer at 0 h and 1 h, respectively.

### 3.5. Measurement of Photophysical Properties

UV–vis absorption and fluorescence spectra were recorded via an absorption spectrometer (UV-1800, Mapada) and a fluorescence spectrophotometer (FP8500, JASCO, Tokyo, Japan) at room-temperature, respectively. The fluorescence lifetime measurements were conducted using a time-correlated single photon counting (TCSPC) apparatus at room temperature and a pulsed laser at a wavelength of 510 nm was used as the excitation source. The nanosecond transient absorption spectra were recorded by a nanosecond flash photolysis system (LP980, Edinburgh instruments, UK) with a pulse laser (7 ns, 1 Hz) from a Nd:YAG laser at a wavelength of 532 nm. The samples in 10 mm path length quartz cuvettes were freshly prepared and deoxygenated by bubbling nitrogen for over 20 min before measurement. The analyzing light was a 450 W pulsed xenon lamp. A monochromator equipped with a photomultiplier for collecting the spectral range from 350 to 850 nm was used to analyze transient absorption spectra. The decay curves were fitted by least-squares regression using a custom-written algorithm in the Matlab.

The corresponding fluorescence quantum yields were calculated by using Equation (3) [59] with (4-((trimethylsilyl)ethynyl)phenyl)-BODIPY as the standard, Φ_F_ = 0.46 in CHCl_3_, *λ*_exc_ 484 nm [53].

(3) $$\Phi_{F}{}_{\left(x\right)}\;=\;\Phi_{F}{}_{\left(\mathrm{std}\right)}\frac{A_{\mathrm{std}}•F_{{}_{x}}}{A_{x}•F_{{}_{\mathrm{std}}}}\;{\left(\frac{n_{x}}{n_{\mathrm{std}}}\right)}^{2}$$

where Φ_F(x)_ and Φ_F(std)_ are the fluorescence quantum yields of the sensitizers and standard, respectively. A_x_ and A_std_ are the absorbance of the sensitizers and standard, F_x_ and F_std_ are the area under the emission curve of the sensitizers and standard, and n is the refractive index of the solvents used in measurement.

## 4. Conclusions

In conclusion, a broadband visible light-absorbing [70]fullerene-BODIPY-triphenylamine triad (**C_70_-B-T**) has been synthesized and used as a heavy atom-free organic triplet photosensitizer for photooxidation. Two TPA units were introduced to the π-core of BODIPY and the absorption spectrum of **C_70_-B-T** covered virtually the entire UV–visible region. Upon the direct or indirect excitation of the BODIPY-part of **C_70_-B-T**, the intramolecular singlet excited state energy transfer from BODIPY to C_70_ unit occurs and produces ^1^C_70_^*^. Then, the ISC of C_70_ produces ^3^C_70_^*^. The photophysical processes were confirmed by steady-state and transient spectroscopies. The photooxidation ability of the photosensitizers was investigated using DHN as a chemical sensor. Among all the investigated compounds, **C_70_-B-T** gives the best photooxidation efficiency. The photooxidation rate constant of **C_70_-B-T** is 1.47 and 1.51 times as that of **C_70_-1** and MB, respectively. The results indicate that C_70_-antenna could be used as another heavy atom-free organic triplet photosensitizer structure motif, with potential applications in photodynamic therapy, photocatalysis, photovoltaics and TTA upconversion.

## Figures and Tables

**Table 1:** Characteristic spectroscopic data of **C_70_-1**, **B-T** and **C_70_-B-T** in toluene and THF *^a^*.

Compound	Solvent	λ_abs_ (nm)	λ_em_ (nm)	Φ_F_ *^b^*	
				Ex. = 339 nm	Ex. = 605 nm
**C_70_-1**	toluene	284, 307, 398, 462, 537, 666	710	-	-
	THF	-	-	-	-
**B-T**	toluene	330, 443, 603	664	0.29	0.22
	THF	-	668	-	0.01
**C_70_-B-T**	toluene	284, 310, 344, 443, 600	664	0.03	0.04
	THF	-	666	-	0.005

*^a^* c = 1.0 × 10^−5^ M. *^b^* Using (4-((trimethylsilyl)ethynyl)phenyl)-BODIPY as reference, Φ_F_ = 0.46 in CHCl_3_, *λ*_exc_ 484 nm [53].

**Table 2:** The photooxidation rate constant and yield of singlet oxygen of the photosensitizers ***^a^***.

Photosensitizers	k_obs_ *^b^*/min^−1^	υ_i_ *^c^*	Φ_∆_ *^d^*
**C_70_-B-T**	67.5	6.75	0.78
**C_70_-1**	45.9	4.59	0.81 ^e^
**B-T**	2.0	0.2	-
MB	44.8	4.48	0.57

*^a^* In CH_2_Cl_2_/CH_3_OH = 9:1 (*v*/*v*). c = 1.0 × 10^−5^ mol/L. *^b^* The rate constant k_obs_ was calculated by the rule: ln[(A − A′)/A_0_] = −k_obs_t. In 10^−3^ min^−1^. *^c^* Initial consumption rate of DHN, υ_i_ = k_obs_[DHN]. In 10^−6^ M min^−1^. *^d^* Quantum yield of singlet oxygen (^1^O_2_), with MB as standard (Φ_∆_ = 0.57 in CH_2_Cl_2_). *^e^* Literature values [32].

## Data Availability

The data presented in this study are available in Supplementary Materials.

## Supplementary Materials

The following are available online: the spectral response of DHN with MB as the sensitizer (Supplementary Figure S1), the photostability of **C_70_-B-T** (Supplementary Figure S2), high resolution mass spectra (Supplementary Figures S3–S7), ^1^H-NMR and ^13^C-NMR spectra (Supplementary Figures S8–S23) are also provided.

## Sample Availability

Samples of the compounds are available from the authors.
