# Supplementary material for: Broadband Visible Light-Absorbing [70]Fullerene-BODIPY-Triphenylamine Triad: Synthesis and Application as Heavy Atom-Free Organic Triplet Photosensitizer for Photooxidation
Source: Molecules. 2021 Feb 25;26(5):1243. doi: 10.3390/molecules26051243 (PMC7956457; doi:10.3390/molecules26051243)
Supplement: Supplementary file 1 [file molecules-26-01243-s001.pdf]

Supplementary Materials

Broadband Visible Light-Absorbing [70]Fullerene-BODIPY-Triphenylamine Triad: Synthesis and Application as Heavy Atom-Free Organic Triplet Photosensitizer for Photooxidation

San-E Zhu,[*1] Jian-Hui Zhang,[1,] Yu Gong,[1,] Li-Feng Dou,[1] Li-Hua Mao,[1] Hong-Dian Lu,[1] Chun-Xiang Wei,[1] Hong Chen,[1] Xue-Fei Wang[*2] and Wei Yang[*1]

[1]School of Energy, Materials and Chemical Engineering, Hefei University, Hefei, Anhui 230601, China.

[2]School of Chemistry and Chemical Engineering, University of Chinese Academy of Sciences, Beijing 100049, China.

E-mail address: zhuse@hfuu.edu.cn (S.-E Z.); wangxf@ucas.ac.cn (X.-F. W.); weyang@ustc.edu.cn (W. Yang).

Contents

1. The spectral response of DHN with MB as the sensitizer S2

2. The photostability of C_{70}-B-T S2

3. High resolution mass spectra S2-7

4. ^{1}H NMR and ^{13}C NMR spectra S7-15

1. The spectral response of DHN with MB as the sensitizer.

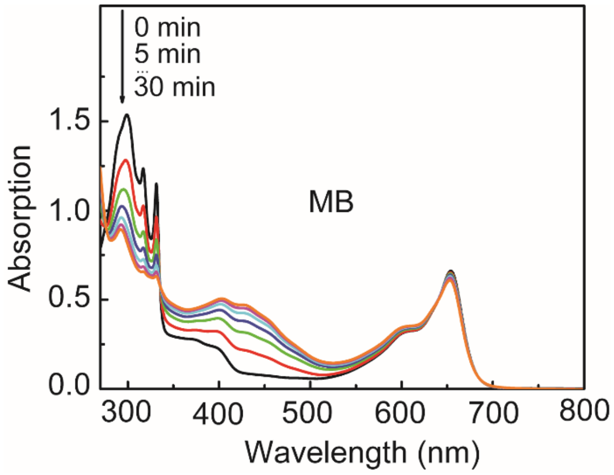

Figure S1. Absorption spectral change for the photooxidation of DHN using **MB** as the sensitizer. c[MB] = 1.0 × 10^{-5} mol L^{-1}, c[DHN] = 1.0 × 10^{-4} mol L^{-1}. In CH_2Cl_2–MeOH (9/1, v/v).

2. The photostability of C_{70}-B-T.

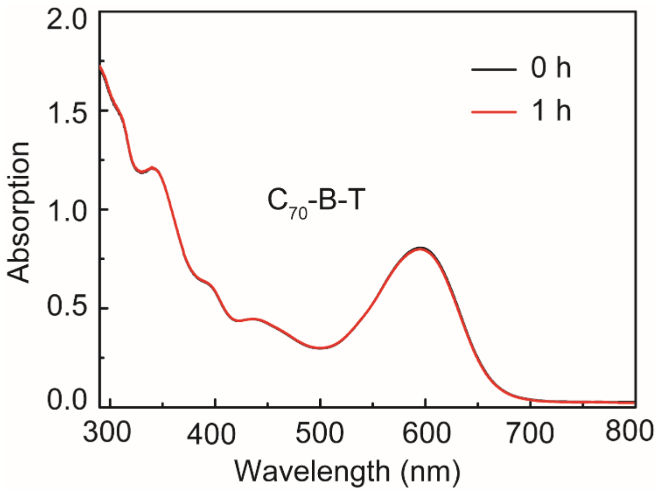

Figure S2. The stability of **C_{70}-B-T**. c = 1.0 × 10^{-5} mol L^{-1} in CH_2Cl_2–MeOH (9/1, v/v). After being exposed to light for 1 h, no bleaching is observed.

3. High resolution mass spectra

D:\data\gc\20201130\ZJH-C70\0_A18\1\1Ref

Comment 1
Comment 2

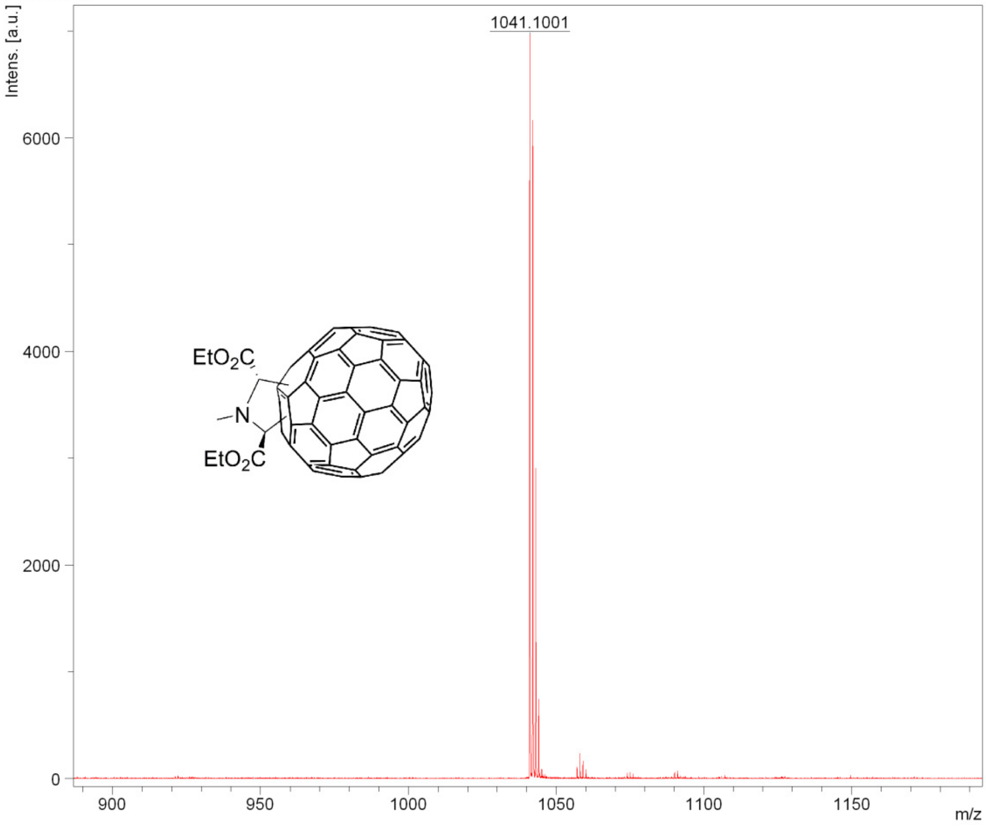

Acquisition Parameter

Date of acquisition	2020-11-30T19:07:21.003+08:00
Acquisition method name	D:\Methods\flexControlMethods\gc-RN_0-1200_Da.par
Aquisition operation mode	Reflector
Voltage polarity	NEG
Number of shots	500
Name of spectrum used for calibration	
Calibration reference list used	sample

Instrument Info

User	BDAL@CN
Instrument	FLEX-PC
Instrument type	ultraflexTOF/TOF

Bruker Daltonics flexAnalysis printed: 2020-11-30 22:17:31

Figure S3. The high resolution mass spectrum (HRMS) of **C_{70}-1**.

D:\data\gc\20201207\ZJH-1\0_G11\1\1Ref

Comment 1

Comment 2

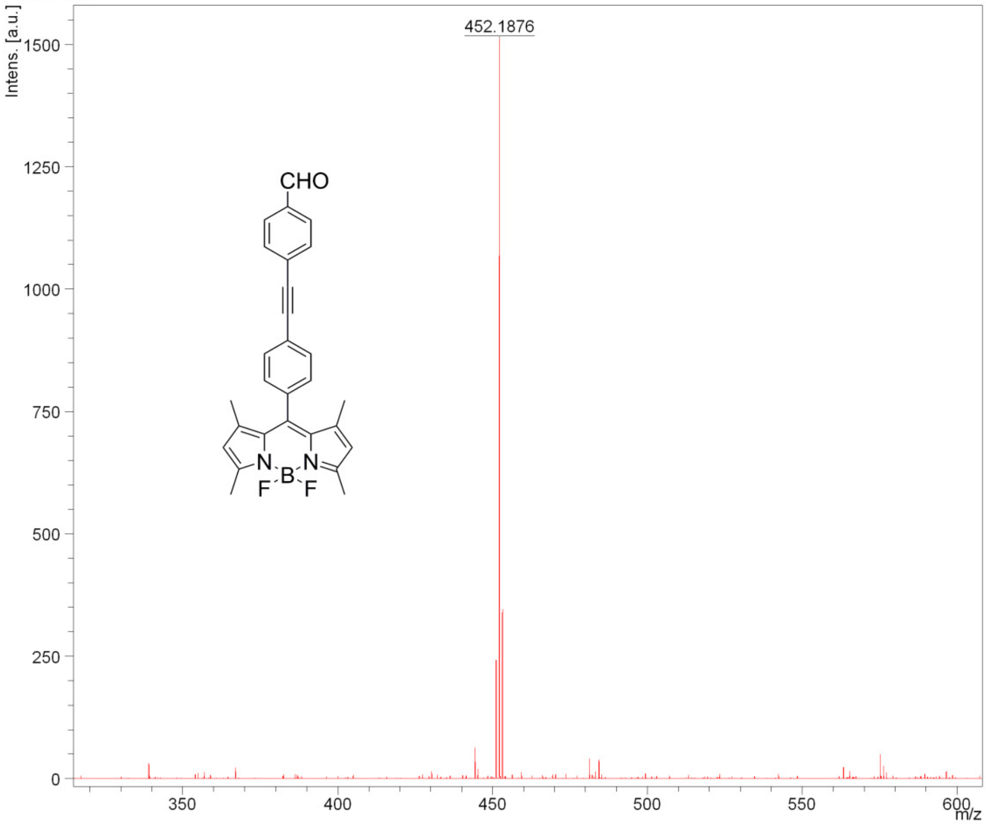

Acquisition Parameter

Date of acquisition	2020-12-07T15:36:14.445+08:00
Acquisition method name	D:\Methods\flexControlMethods\gc-RP_100-1500_Da.par
Aquisition operation mode	Reflector
Voltage polarity	POS
Number of shots	500
Name of spectrum used for calibration	
Calibration reference list used	sample

Instrument Info

User	BDAL@CN
Instrument	FLEX-PC
Instrument type	ultraflexTOF/TOF

Bruker Daltonics flexAnalysis printed: 2020-12-7 16:27:32

Figure S4. The high resolution mass spectrum (HRMS) of **5**.

D:\data\gc\20201207\ZJH-2\0_G12\1\1Ref

Comment 1

Comment 2

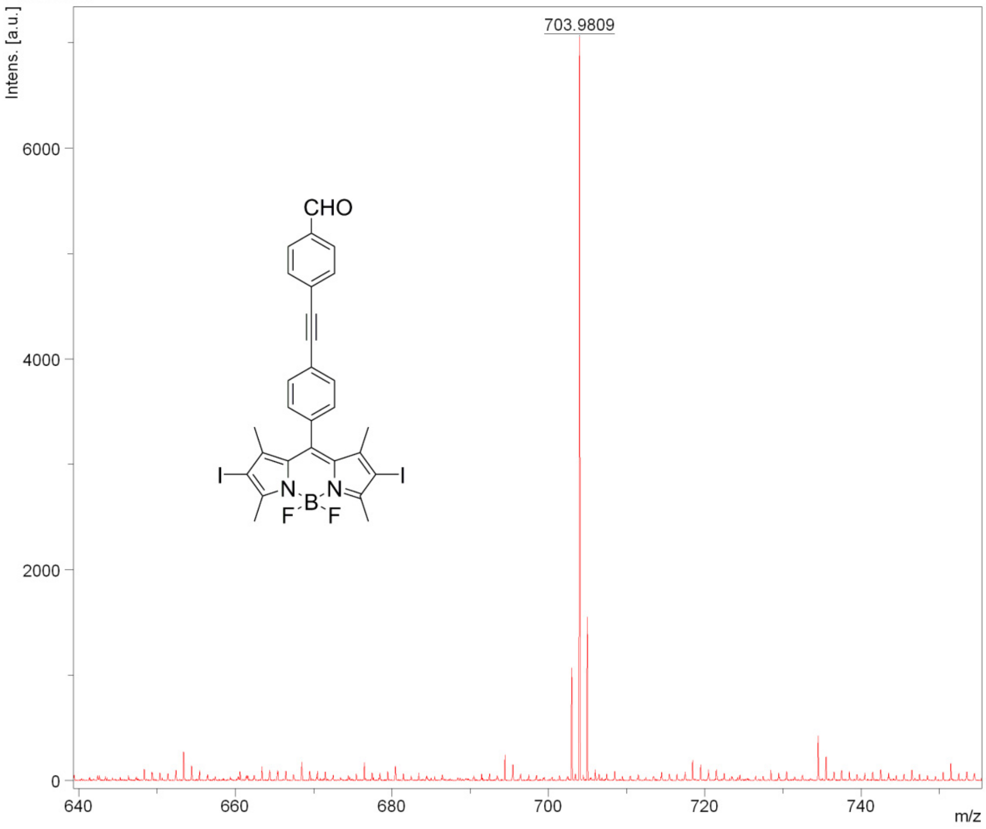

Acquisition Parameter

Date of acquisition	2020-12-07T15:38:11.898+08:00
Acquisition method name	D:\Methods\flexControlMethods\gc-RP_100-1500_Da.par
Aquisition operation mode	Reflector
Voltage polarity	POS
Number of shots	500
Name of spectrum used for calibration	
Calibration reference list used	sample

Instrument Info

User	BDAL@CN
Instrument	FLEX-PC
Instrument type	ultraflexTOF/TOF

Bruker Daltonics flexAnalysis printed: 2020-12-7 16:28:47

Figure S5. The high resolution mass spectrum (HRMS) of **6**.

D:\data\gc\20201130\ZJH-BDP-2Ph\0_A23\1\1Ref

Comment 1
Comment 2

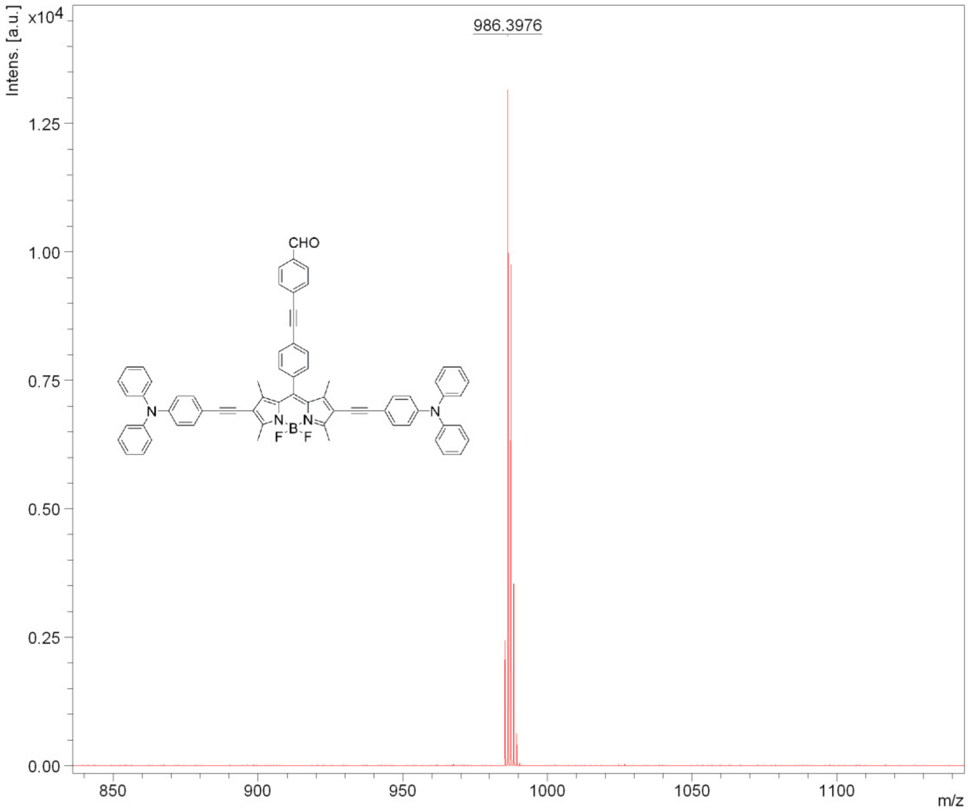

Acquisition Parameter

Date of acquisition	2020-11-30T19:19:45.159+08:00
Acquisition method name	D:\Methods\flexControlMethods\gc-RP_100-1500_Da.par
Aquisition operation mode	Reflector
Voltage polarity	POS
Number of shots	500
Name of spectrum used for calibration	
Calibration reference list used	sample

Instrument Info

User	BDAL@CN
Instrument	FLEX-PC
Instrument type	ultraflexTOF/TOF

Bruker Daltonics flexAnalysis printed: 2020-11-30 22:26:19

Figure S6. The high resolution mass spectrum (HRMS) of **B-T**.

D:\data\gc\20201216\ZJH-201214-670\0_H20\1\1Ref

Comment 1
Comment 2

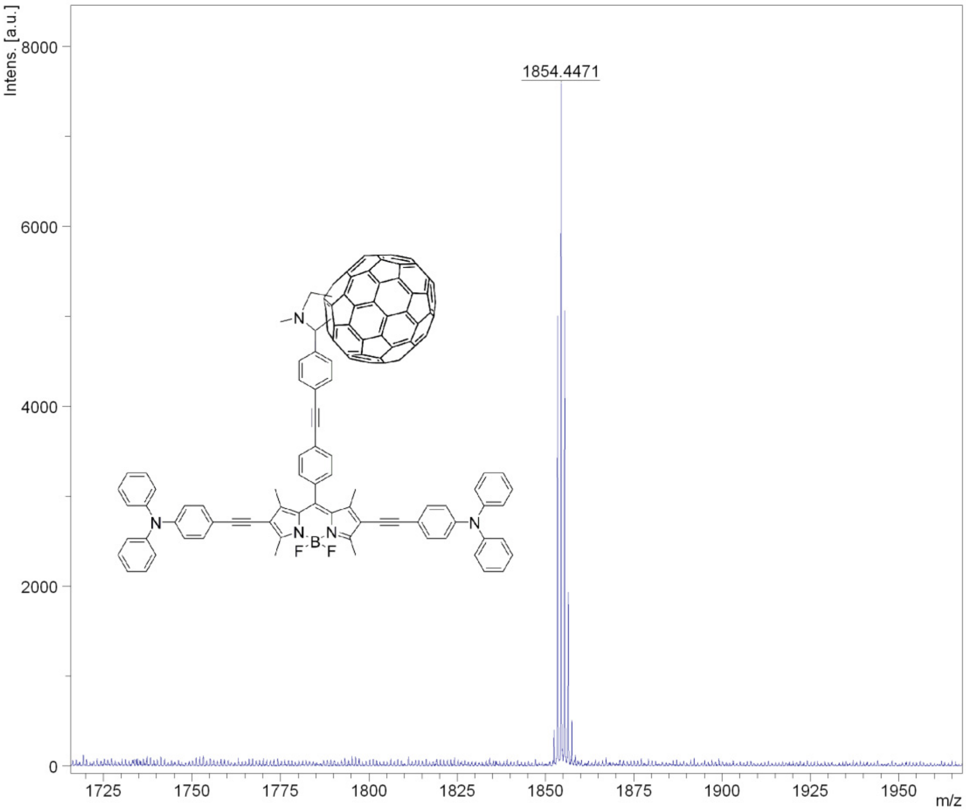

Acquisition Parameter

Date of acquisition	2020-12-16T20:47:47.423+08:00
Acquisition method name	D:\Methods\flexControlMethods\gc-RP_100-1500_Da.par
Aquisition operation mode	Reflector
Voltage polarity	POS
Number of shots	500
Name of spectrum used for calibration	
Calibration reference list used	sample

Instrument Info

User	BDAL@CN
Instrument	FLEX-PC
Instrument type	ultraflexTOF/TOF

Bruker Daltonics flexAnalysis printed: 2020-12-17 17:07:15

Figure S7. The high resolution mass spectrum (HRMS) of **C_{70}-B-T**.

4. ^{1}H NMR and ^{13}C NMR spectra

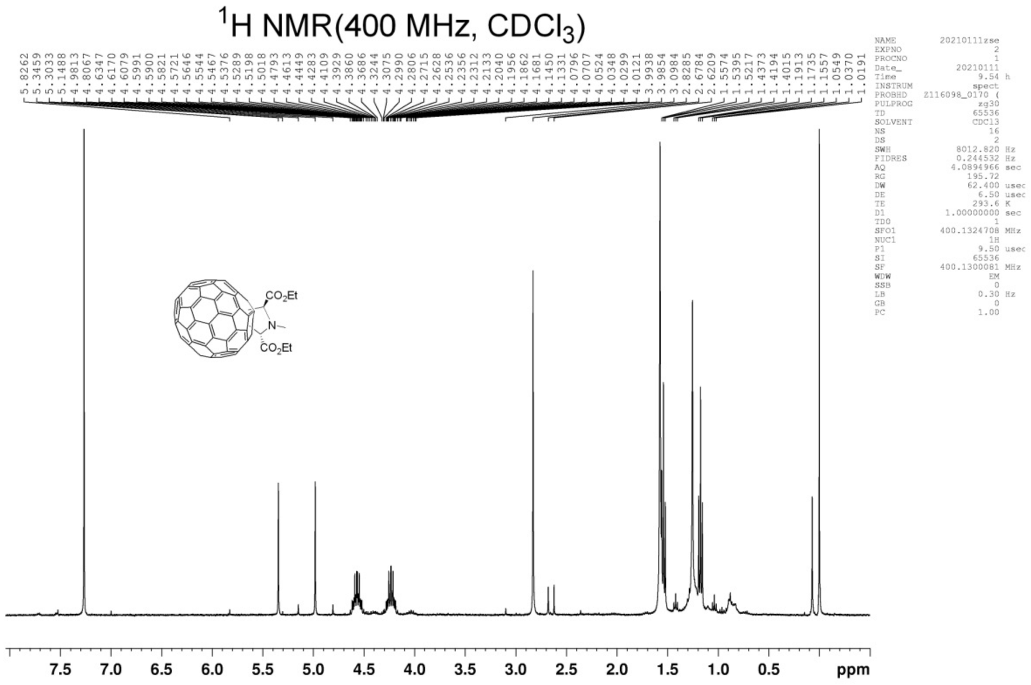

Figure S8. ^{1}H NMR of $\mathbf{C_{70}}$**-1** in $CDCl_3$ (400 MHz).

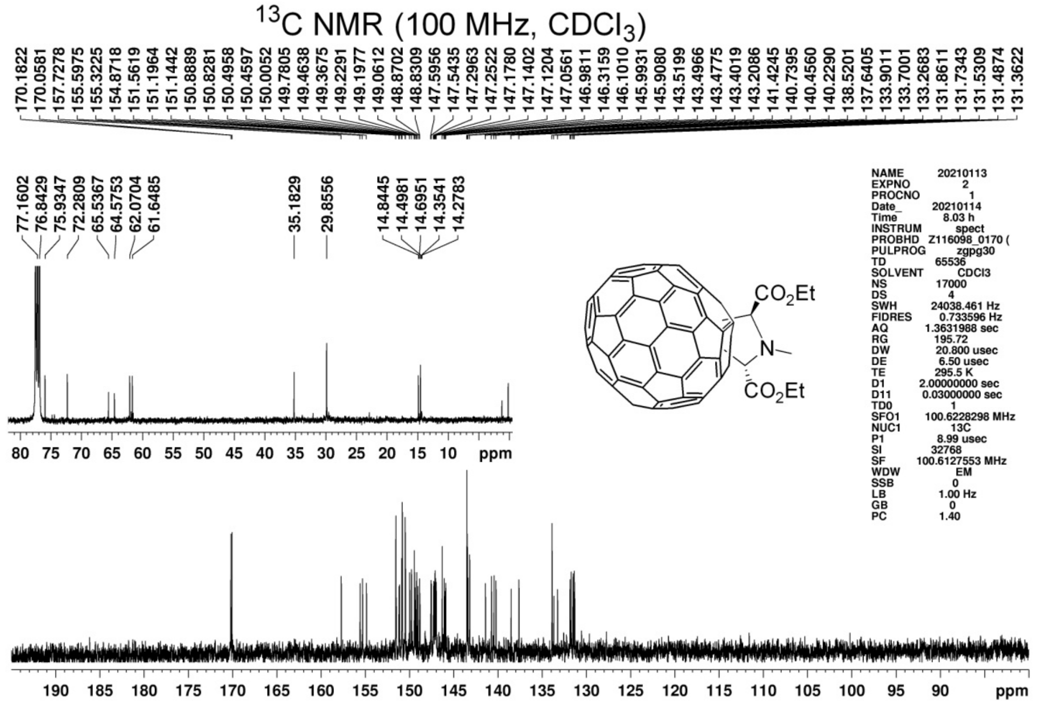

Figure S9. ^{13}C NMR of $\mathbf{C_{70}}$**-1** in $CDCl_3$ (100 MHz).

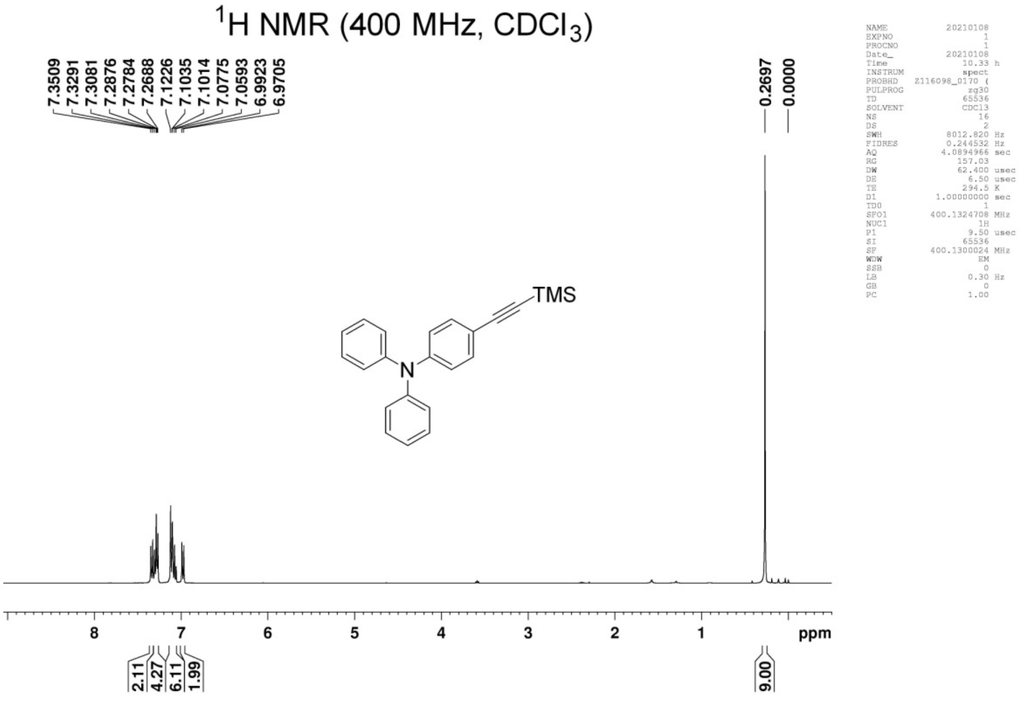

Figure S10. ^{1}H NMR of **2** in $CDCl_3$ (400 MHz).

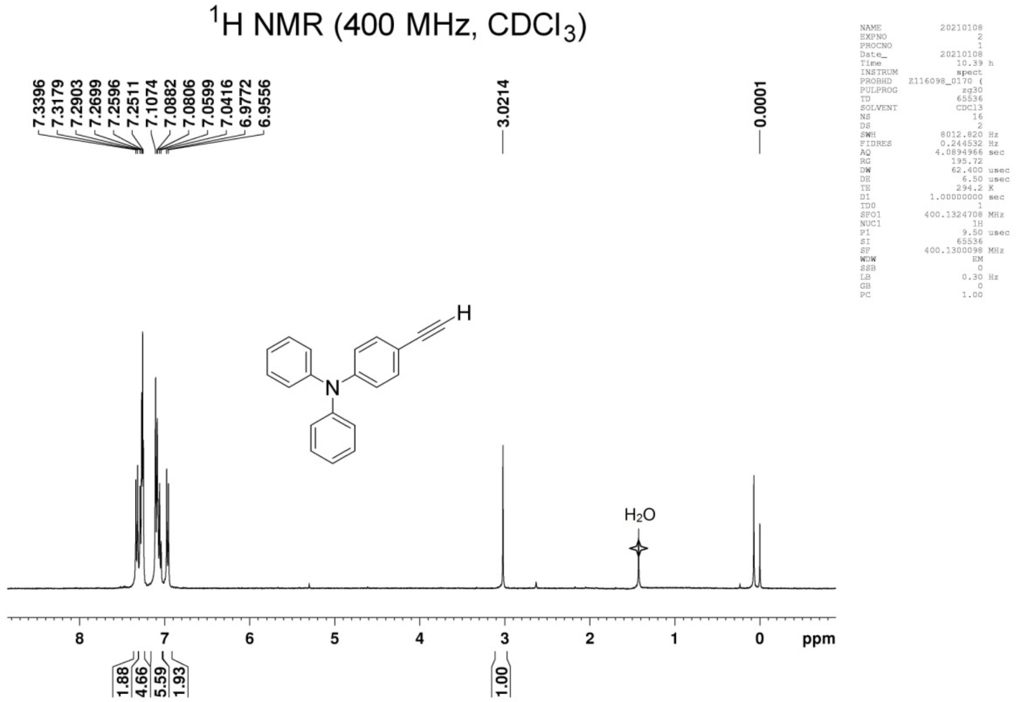

Figure S11. ^{1}H NMR of **3** in $CDCl_3$ (400 MHz).

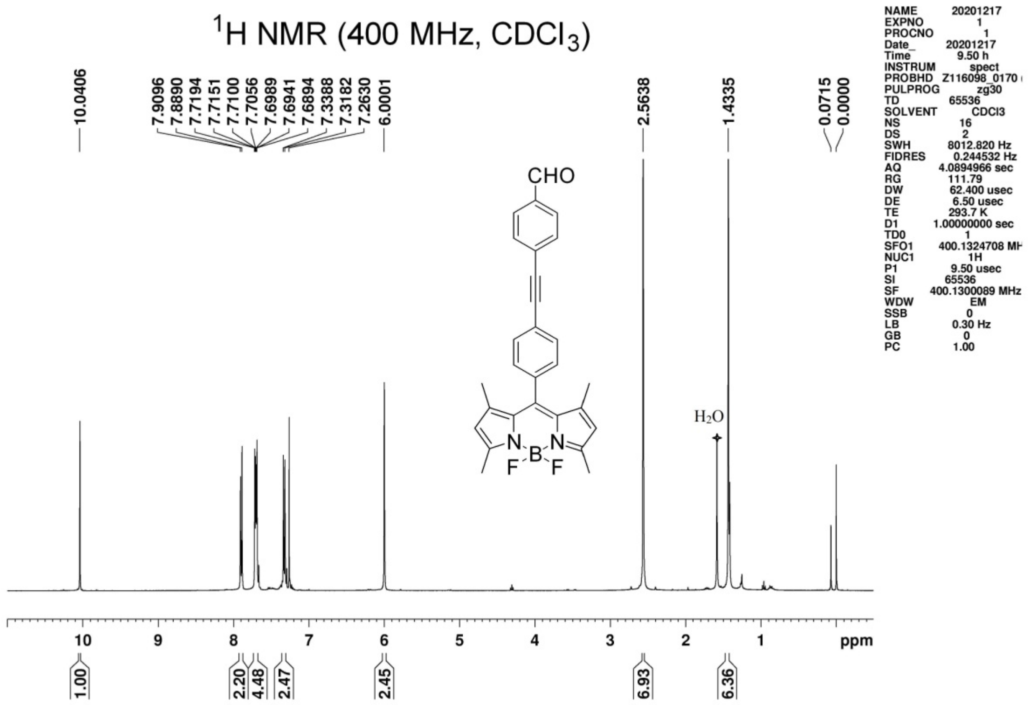

Figure S12. ^{1}H NMR of **5** in $CDCl_3$ (400 MHz).

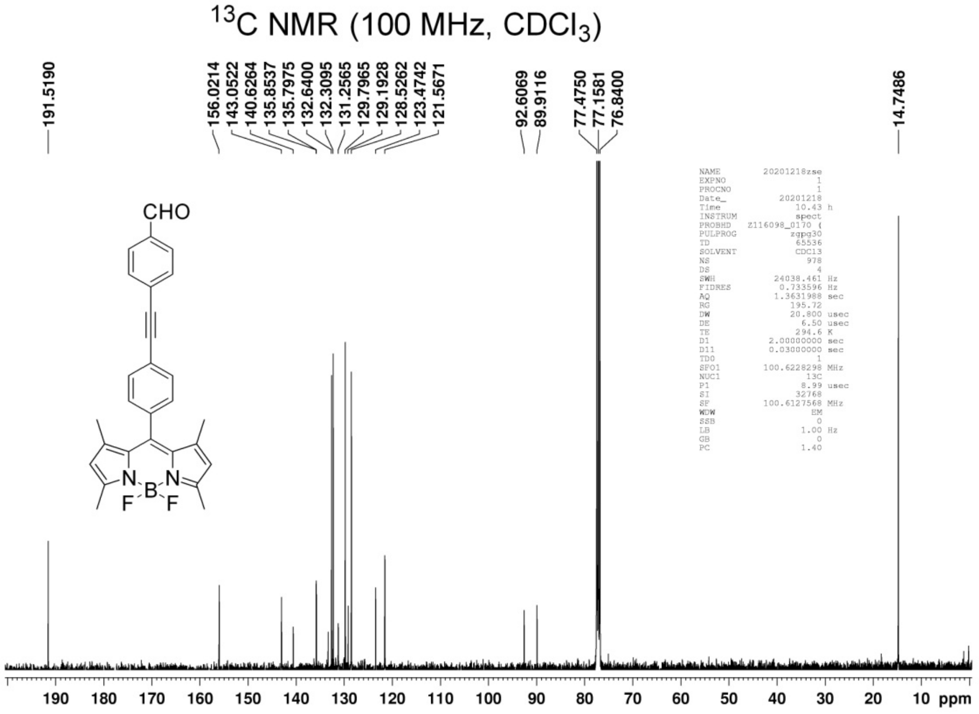

Figure S13. ^{13}C NMR of **5** in $CDCl_3$ (100 MHz).

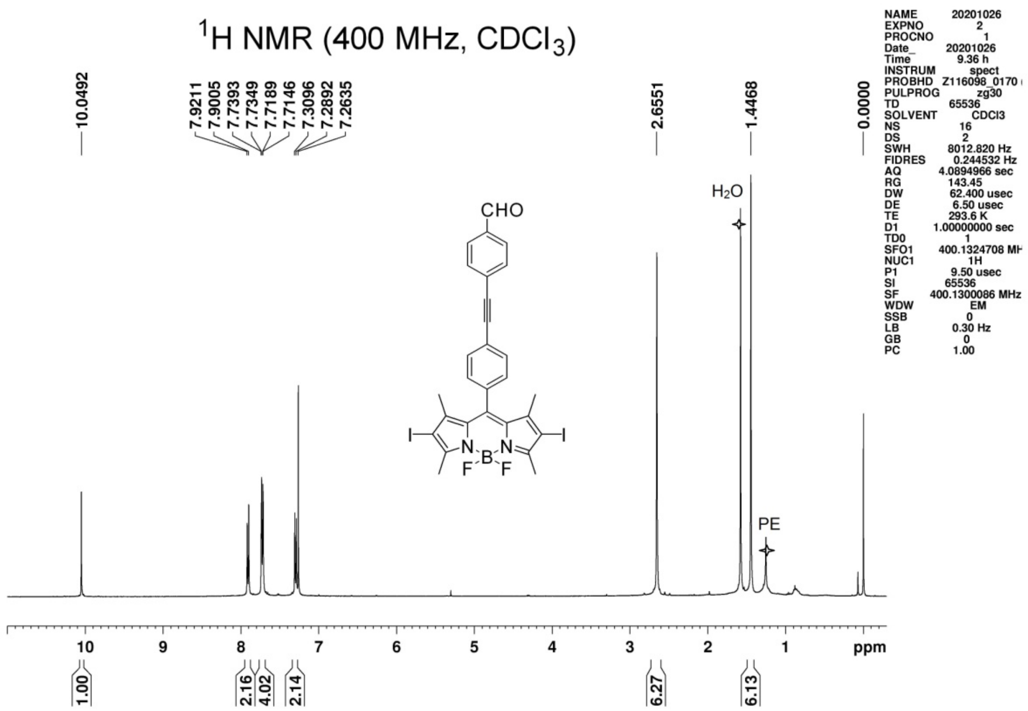

Figure S14. ^{1}H NMR of **6** in $CDCl_3$ (400 MHz).

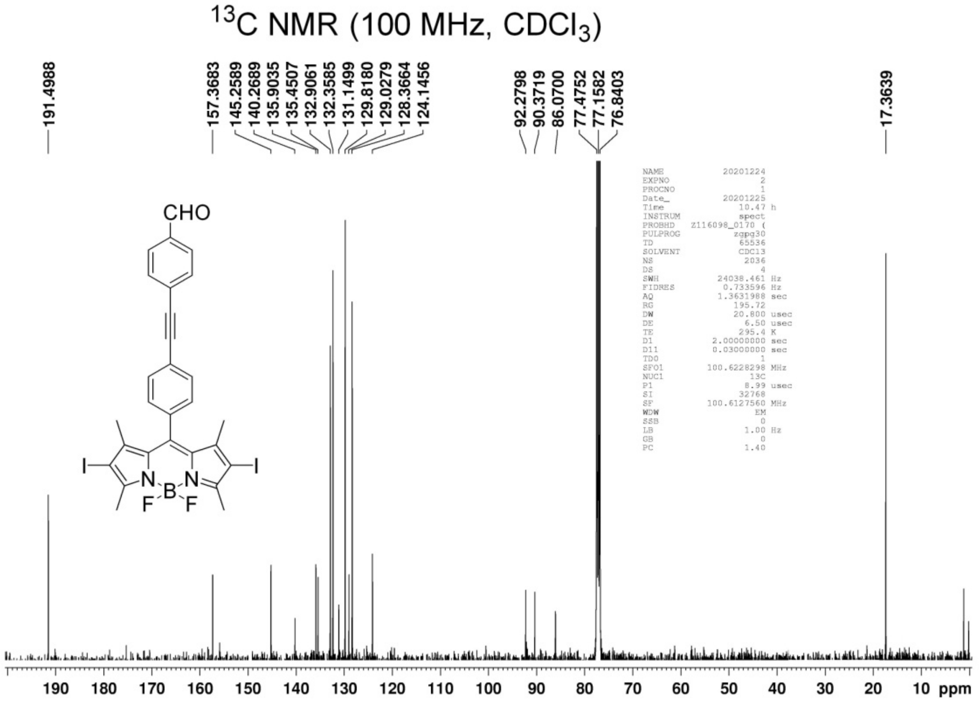

Figure S15. ^{13}C NMR of **6** in $CDCl_3$ (100 MHz).

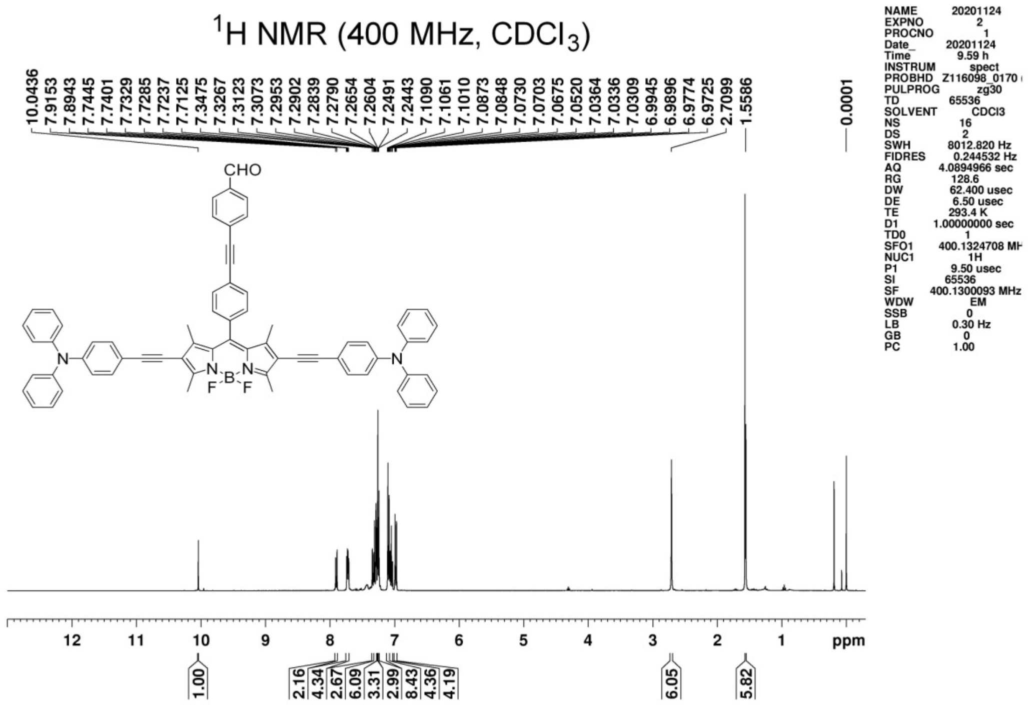

Figure S16. ^{1}H NMR of **B-T** in CDCl$_3$ (400 MHz).

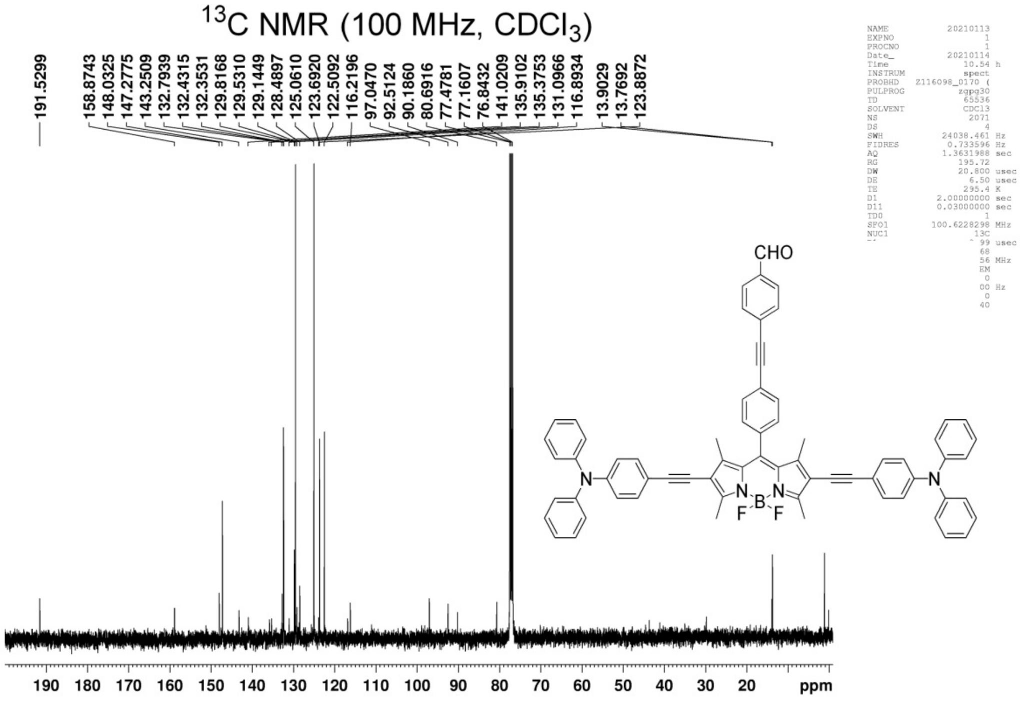

Figure S17. ^{13}C NMR of **B-T** in CDCl$_3$ (100 MHz).

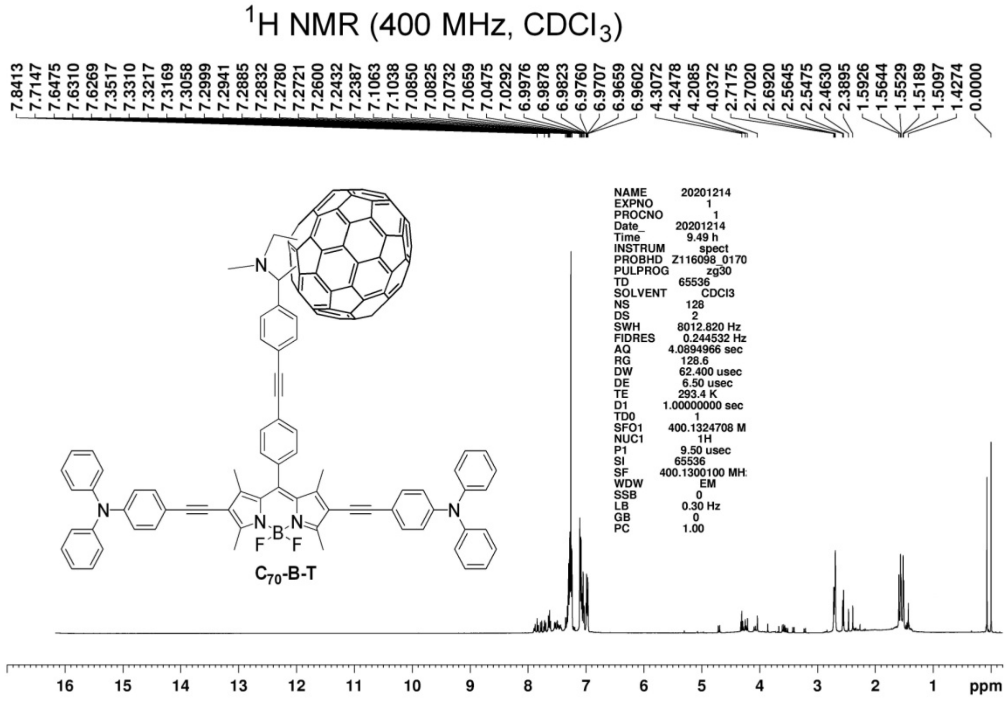

Figure S18. ^{1}H NMR of **C_{70}-B-T** in $CDCl_3$ (400 MHz).

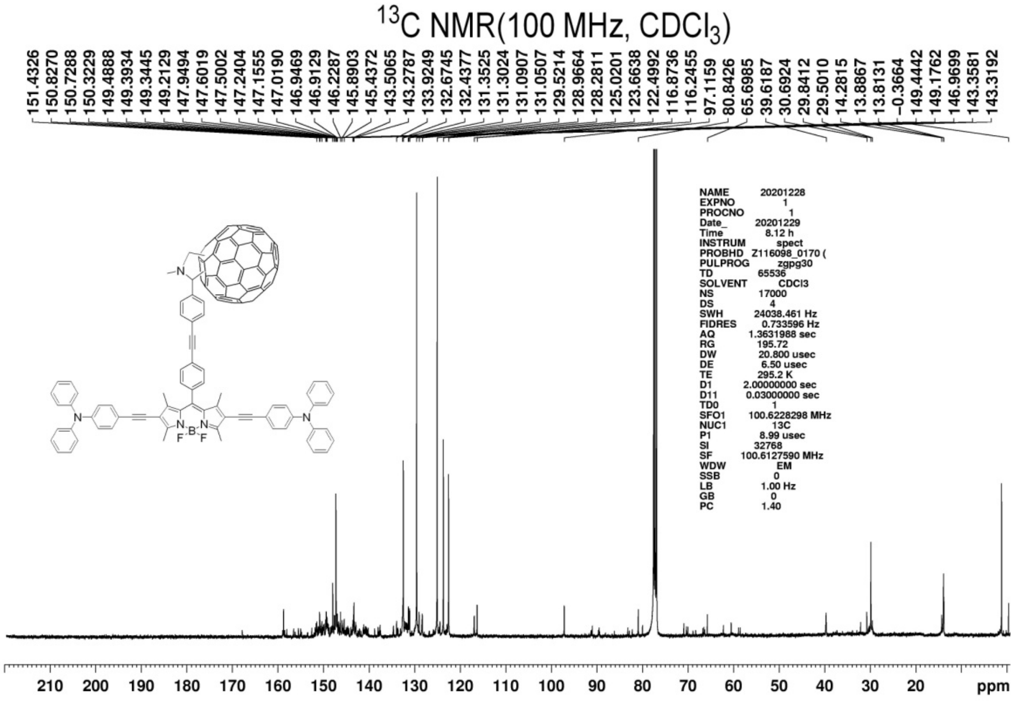

Figure S19. ^{13}C NMR of **C_{70}-B-T** in $CDCl_3$ (100 MHz).

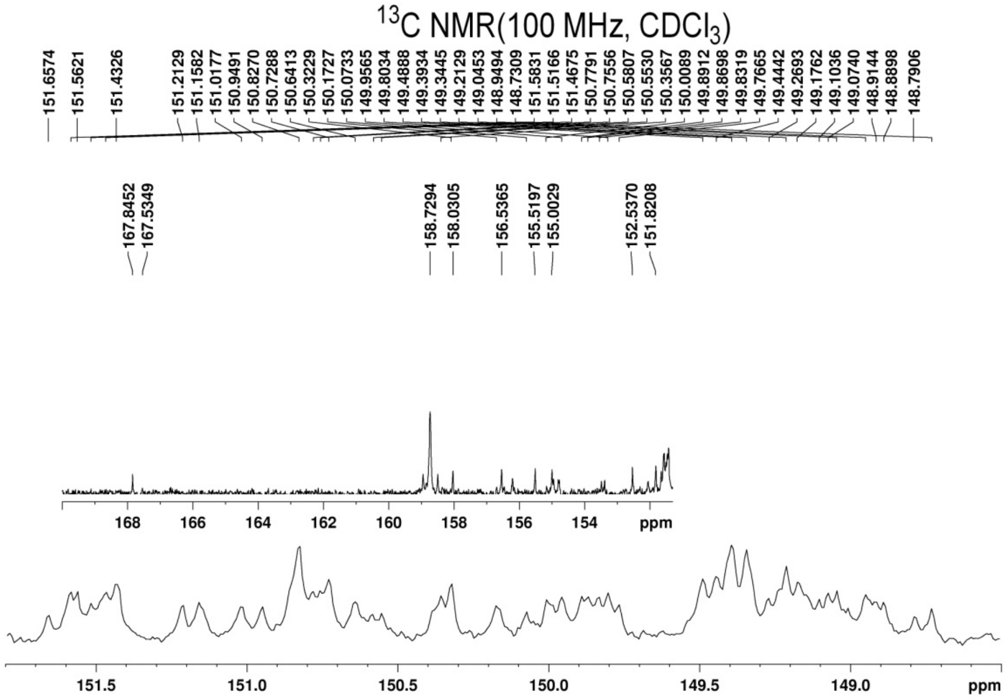

Figure S20. Expansion of the ^{13}C NMR of **C_{70}-B-T**.

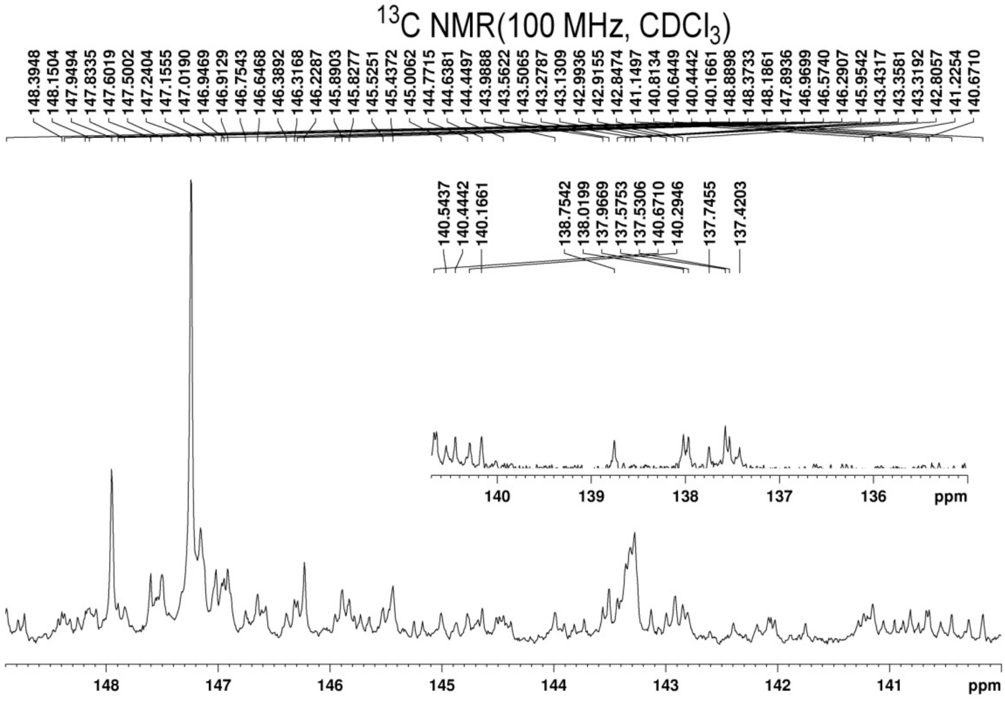

Figure S21. Expansion of the ^{13}C NMR of **C_{70}-B-T**.

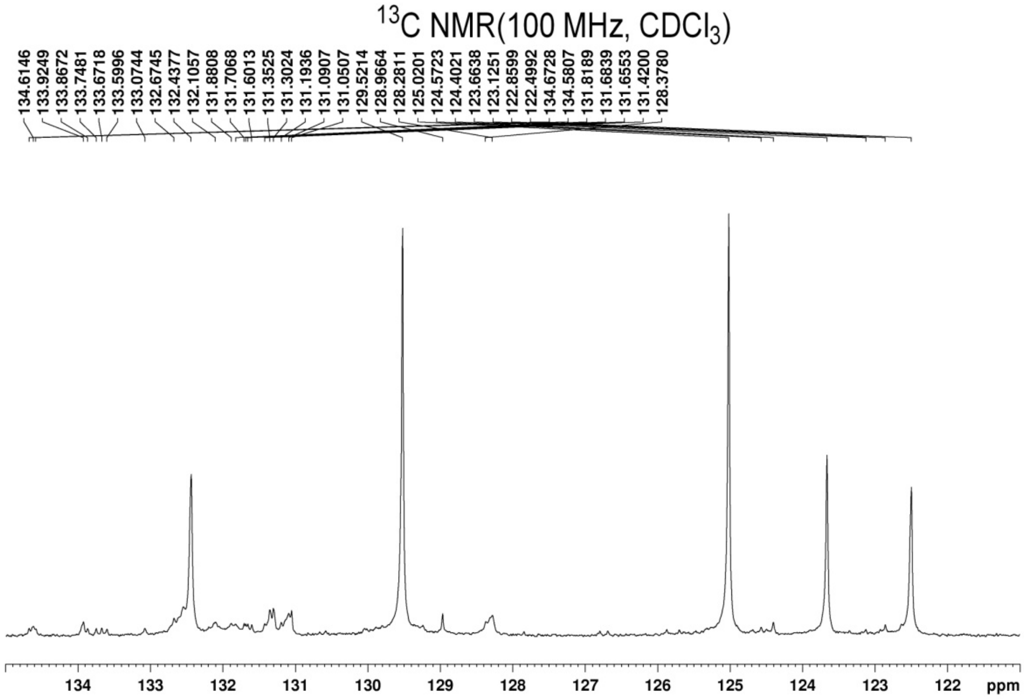

Figure S22. Expansion of the ^{13}C NMR of **C_{70}-B-T**.

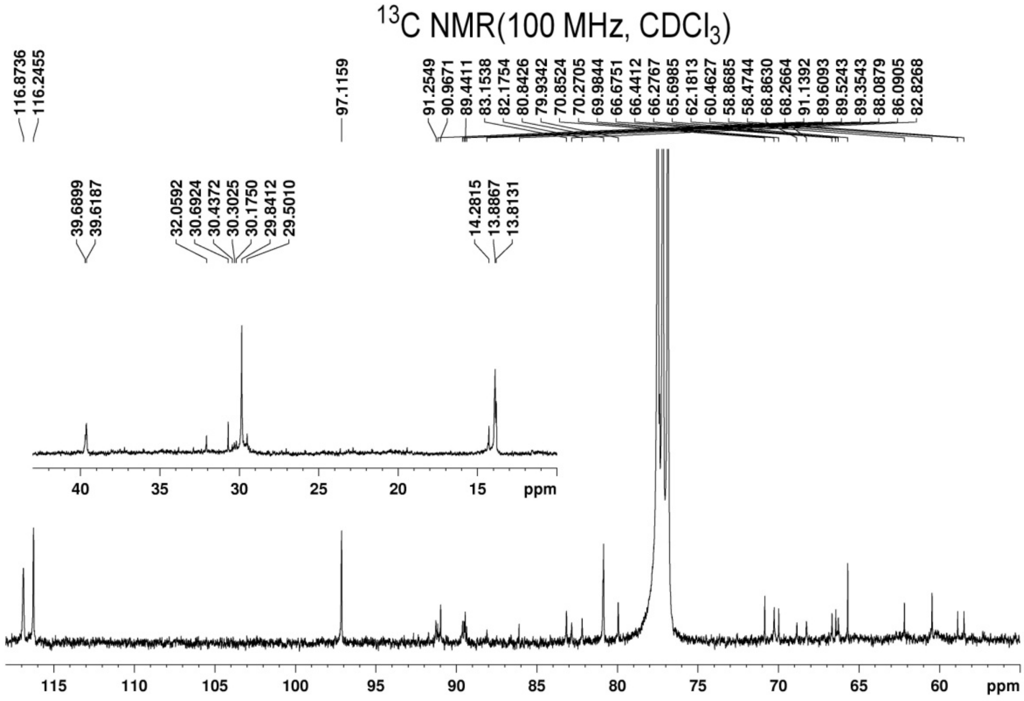

Figure S23. Expansion of the ^{13}C NMR of **C_{70}-B-T**.
